# Supplementary material for: The application of allostasis and allostatic load in animal species: A scoping review
Source: PLoS One. 2022 Aug 30;17(8):e0273838. doi: 10.1371/journal.pone.0273838 (PMC9426905; doi:10.1371/journal.pone.0273838)
Supplement: S1 Dataset — (DOCX) [file pone.0273838.s002.docx]

**S1 Dataset:** Full reference list of 572 articles included in the scoping review

1. Abbink W, Bevelander GS, Rotllant J, Canario AVM, Flik G. Calcium handling in Sparus auratus: effects of water and dietary calcium levels on mineral composition, cortisol and PTHrP levels. J Exp Biol. 2004;207: 4077–4084.

2. Adamo SA, Baker JL. Conserved features of chronic stress across phyla: The effects of long-term stress on behavior and the concentration of the neurohormone octopamine in the cricket, Gryllus texensis. Horm Behav. 2011;60: 478–483. doi:10.1016/j.yhbeh.2011.07.015

3. Adams DB, Thornber PM. Epidemiology, ethics and managing risks for physiological and behavioural stability of animals during long distance transportation. Adams DB, Thornber PM, editors. Vet Ital. 2008;44: 165–176.

4. Addis EA, Davis JE, Miner BE, Wingfield JC. Variation in circulating corticosterone levels is associated with altitudinal range expansion in a passerine bird. Oecologia. 2011;167: 369–378. doi:10.1007/s00442-011-2001-5

5. Aerts J, Schaeck M, De Swaef E, Ampe B, Decostere A. Vibrio lentus as a probiotic candidate lowers glucocorticoid levels in gnotobiotic sea bass larvae. Aquaculture. 2018;492: 40–45. doi:10.1016/j.aquaculture.2018.03.059

6. Ågren G, Lund I, Thiblin I, Lundeberg T. Tail skin temperatures reflect coping styles in rats. Physiol Behav. 2009;96: 374–382. doi:10.1016/j.physbeh.2008.11.003

7. Aidos L, Cafiso A, Serra V, Vasconi M, Bertotto D, Bazzocchi C, et al. How Different Stocking Densities Affect Growth and Stress Status of Acipenser baerii Early Stage Larvae. Animals. 2020;10: 1289. doi:10.3390/ani10081289

8. Alagawany M, Farag MR, Abd El-Hack ME, Dhama K, Fowler J. Use of acetylsalicylic acid as a feed additive in poultry nutrition. Worlds Poult Sci J. 2017;73: 633–642. doi:10.1017/S0043933917000253

9. Alfonso-Avila AR, Charbonneau E, Chouinard PY, Tremblay GF, Gervais R. Potassium carbonate as a cation source for early-lactation dairy cows fed high-concentrate diets. J Dairy Sci. 2017;100: 1751–1765. doi:10.3168/jds.2016-11776

10. Anderson L, Cree A, Towns D, Nelson N. Modulation of corticosterone secretion in tuatara (Sphenodon punctatus): Evidence of a dampened stress response in gravid females. Gen Comp Endocrinol. 2014;201: 45–52. doi:10.1016/j.ygcen.2014.03.035

11. Anderson L, Nelson N, Cree A. Glucocorticoids in tuatara (Sphenodon punctatus): Some influential factors, and applications in conservation management. Gen Comp Endocrinol. 2017;244: 54–59. doi:10.1016/j.ygcen.2015.12.001

12. Anderson PA, Berzins IK, Fogarty F, Hamlin HJ, Guillette LJ. Sound, stress, and seahorses: The consequences of a noisy environment to animal health. Aquaculture. 2011;311: 129–138. doi:10.1016/j.aquaculture.2010.11.013

13. Åberg Andersson M, Silva PIM, Steffensen JF, Höglund E. Effects of maternal stress coping style on offspring characteristics in rainbow trout (Oncorhynchus mykiss). Horm Behav. 2011;60: 699–705. doi:10.1016/j.yhbeh.2011.09.008

14. Anestis SF. Urinary cortisol responses to unusual events in captive chimpanzees ( *Pan troglodytes* ). Stress. 2009;12: 49–57. doi:10.1080/10253890802041308

15. Angelier F, Chastel O. Stress, prolactin and parental investment in birds: A review. Gen Comp Endocrinol. 2009;163: 142–148. doi:10.1016/j.ygcen.2009.03.028

16. Angelier F, Clementchastel C, Gabrielsen G, Chastel O. Corticosterone and time–activity budget: An experiment with Black-legged kittiwakes. Horm Behav. 2007;52: 482–491. doi:10.1016/j.yhbeh.2007.07.003

17. Angelier F, Clement-Chastel C, Welcker J, Gabrielsen GW, Chastel O. How does corticosterone affect parental behaviour and reproductive success? A study of prolactin in black-legged kittiwakes. Funct Ecol. 2009;23: 784–793. doi:10.1111/j.1365-2435.2009.01545.x

18. Angelier F, Costantini D, Blevin P, Chastel O. Do glucocorticoids mediate the link between environmental conditions and telomere dynamics in wild vertebrates? A review. Gen Comp Endocrinol. 2018;256: 99–111. doi:10.1016/j.ygcen.2017.07.007

19. Angelier F, Giraudeau M, Bost C-A, Le Bouard F, Chastel O. Are stress hormone levels a good proxy of foraging success? An experiment with King Penguins, Aptenodytes patagonicus. J Exp Biol. 2009;212: 2824–2829. doi:10.1242/jeb.027722

20. Angelier F, Meillère A, Grace JK, Trouvé C, Brischoux F. No evidence for an effect of traffic noise on the development of the corticosterone stress response in an urban exploiter. Gen Comp Endocrinol. 2016;232: 43–50. doi:10.1016/j.ygcen.2015.12.007

21. Angelier F, Parenteau C, Ruault S, Angelier N. Endocrine consequences of an acute stress under different thermal conditions: a study of corticosterone, prolactin, and thyroid hormones in the pigeon (Columbia livia). Comp Biochem Physiol Mol Integr Physiol. 2016;196: 38–45. doi:10.1016/j.cbpa.2016.02.010

22. Angelier F, Parenteau C, Trouvé C, Angelier N. The behavioural and physiological stress responses are linked to plumage coloration in the rock pigeon (Columbia livia). Physiol Behav. 2018;184: 261–267. doi:10.1016/j.physbeh.2017.12.012

23. Angelier F, Shaffer SA, Weimerskirch H, Trouvé C, Chastel O. Corticosterone and Foraging Behavior in a Pelagic Seabird. Physiol Biochem Zool. 2007;80: 283–292. doi:10.1086/512585

24. Angelier F, Vleck CM, Holberton RL, Marra PP. Telomere length, non‐breeding habitat and return rate in male A merican redstarts. Blount J, editor. Funct Ecol. 2013;27: 342–350. doi:10.1111/1365-2435.12041

25. Angelier F, Wingfield JC, Tartu S, Chastel O. Does prolactin mediate parental and life-history decisions in response to environmental conditions in birds? A review. Horm Behav. 2016;77: 18–29. doi:10.1016/j.yhbeh.2015.07.014

26. Angelier F, Wingfield JC, Trouvé C, de Grissac S, Chastel O. Modulation of the prolactin and the corticosterone stress responses: Do they tell the same story in a long-lived bird, the Cape petrel? Gen Comp Endocrinol. 2013;182: 7–15. doi:10.1016/j.ygcen.2012.10.008

27. Angelier F, Wingfield JC, Weimerskirch H, Chastel O. Hormonal correlates of individual quality in a long-lived bird: a test of the “corticosterone-fitness hypothesis.” Biol Lett. 2010;6: 846–849. doi:10.1098/rsbl.2010.0376

28. Angelier F, Wingfield JC. Importance of the glucocorticoid stress response in a changing world: Theory, hypotheses and perspectives. Gen Comp Endocrinol. 2013;190: 118–128. doi:10.1016/j.ygcen.2013.05.022

29. Arjona FJ, Vargas-Chacoff L, Martín del Río MP, Flik G, Mancera JM, Klaren PHM. The involvement of thyroid hormones and cortisol in the osmotic acclimation of Solea senegalensis. Gen Comp Endocrinol. 2008;155: 796–803. doi:10.1016/j.ygcen.2007.09.007

30. Arjona FJ, Vargas-Chacoff L, Ruiz-Jarabo I, Gonçalves O, Páscoa I, Martín del Río MP, et al. Tertiary stress responses in Senegalese sole (Solea senegalensis Kaup, 1858) to osmotic challenge: Implications for osmoregulation, energy metabolism and growth. Aquaculture. 2009;287: 419–426. doi:10.1016/j.aquaculture.2008.10.047

31. Arlettaz R, Nusslé S, Baltic M, Vogel P, Palme R, Jenni-Eiermann S, et al. Disturbance of wildlife by outdoor winter recreation: allostatic stress response and altered activity–energy budgets. Ecol Appl. 2015;25: 1197–1212. doi:10.1890/14-1141.1

32. Arlettaz R, Patthey P, Baltic M, Leu T, Schaub M, Palme R, et al. Spreading free-riding snow sports represent a novel serious threat for wildlife. Proc R Soc B Biol Sci. 2007;274: 1219–1224. doi:10.1098/rspb.2006.0434

33. Arnason T, Gunnarsson A, Steinarsson A, Danielsdottir AK, Bjornsson BT. Impact of temperature and growth hormone on growth physiology of juvenile Atlantic wolffish (Anarhichas lupus). Aquaculture. 2019;504: 404–413. doi:10.1016/j.aquaculture.2019.02.025

34. Arora B, Jai-Chyi Pei K, Feng Weng C, Ching-Min Sun N. Measuring fecal metabolites of endogenous steroids using ESI-MS/MS spectra in Taiwanese pangolin, (order Pholidota, family Manidae, Genus: Manis): A non-invasive method for endangered species. Gen Comp Endocrinol. 2020;299: 113607. doi:10.1016/j.ygcen.2020.113607

35. Arts JWM, Kramer K, Arndt SS, Ohl F. Sex Differences in Physiological Acclimatization after Transfer in Wistar Rats. Animals. 2014;4: 693–711. doi:10.3390/ani4040693

36. Arts JWM, Oosterhuis NR, Kramer K, Ohl F. Effects of Transfer from Breeding to Research Facility on the Welfare of Rats. Animals. 2014;4: 712–728. doi:10.3390/ani4040712

37. Azevedo A, Wauters J, Kirschbaum C, Serra R, Rivas A, Jewgenow K. Sex steroids and glucocorticoid ratios in Iberian lynx hair. Conserv Physiol. 2020;8. doi:10.1093/conphys/coaa075

38. Bakaloudis DE, Papakosta MA, Guy-Yosef M, Kosicki JZ, Goutner V, Vlachos CG, et al. Sibling competition affects body condition and allostatic load in the colonial nesting lesser kestrel Falco naumanni. J Vertebr Biol. 2020;69: 19058. doi:10.25225/jvb.19058

39. Balasch JC, Tort L. Netting the Stress Responses in Fish. Front Endocrinol. 2019;10: 62. doi:10.3389/fendo.2019.00062

40. Baldo MB, Luna F, Schleich CE, Antenucci CD. Thermoregulatory development and behavior of Ctenomys talarum pups during brief repeated postnatal isolation. Comp Biochem Physiol A Mol Integr Physiol. 2014;173: 35–41. doi:10.1016/j.cbpa.2014.03.008

41. Ballester-Lozano GF, Benedito-Palos L, Navarro JC, Kaushik S, Pérez-Sánchez J. Prediction of fillet fatty acid composition of market-size gilthead sea bream (Sparus aurata) using a regression modelling approach. Aquaculture. 2011;319: 81–88. doi:10.1016/j.aquaculture.2011.06.015

42. Barsotti AMG, Madelaire CB, Wagener C, Titon Jr B, Measey J, Gomes FR. Challenges of a novel range: Water balance, stress, and immunity in an invasive toad. Comp Biochem Physiol A Mol Integr Physiol. 2021;253: 110870. doi:10.1016/j.cbpa.2020.110870

43. Bartolomucci A, Palanza P, Costoli T, Savani E, Laviola G, Parmigiani S, et al. Chronic psychosocial stress persistently alters autonomic function and physical activity in mice. Physiol Behav. 2003;80: 57–67. doi:10.1016/S0031-9384(03)00209-9

44. Basile F, Capaccia C, Zampini D, Biagetti T, Diverio S, Guelfi G. Omics Insights into Animal Resilience and Stress Factors. Animals. 2020;11: 47. doi:10.3390/ani11010047

45. Bauch C, Riechert J, Verhulst S, Becker PH. Telomere length reflects reproductive effort indicated by corticosterone levels in a long-lived seabird. Mol Ecol. 2016;25: 5785–5794. doi:10.1111/mec.13874

46. Bauer CM, Hayes LD, Ebensperger LA, Romero LM. Seasonal variation in the degu (Octodon degus) endocrine stress response. Gen Comp Endocrinol. 2014;197: 26–32. doi:10.1016/j.ygcen.2013.11.025

47. Bauer CM, Skaff NK, Bernard AB, Trevino JM, Ho JM, Romero LM, et al. Habitat type influences endocrine stress response in the degu (Octodon degus). Gen Comp Endocrinol. 2013;186: 136–144. doi:10.1016/j.ygcen.2013.02.036

48. Beehner JC, Bergman TJ. The next step for stress research in primates: To identify relationships between glucocorticoid secretion and fitness. Horm Behav. 2017;91: 68–83. doi:10.1016/j.yhbeh.2017.03.003

49. Beerda B, Ouweltjes W, Sebek LBJ, Windig JJ, Veerkamp RF. Effects of genotype by environment interactions on milk yield, energy balance, and protein balance. J Dairy Sci. 2007;90: 219–228. doi:10.3168/jds.S0022-0302(07)72623-1

50. Belden LK, Rubbo MJ, Wingfield JC, Kiesecker JM. Searching for the Physiological Mechanism of Density Dependence: Does Corticosterone Regulate Tadpole Responses to Density? Physiol Biochem Zool. 2007;80: 444–451. doi:10.1086/518375

51. Belle SV, Estrada A, Ziegler TE, Strier KB. Social and hormonal mechanisms underlying male reproductive strategies in black howler monkeys (Alouatta pigra). Horm Behav. 2009;56: 355–363. doi:10.1016/j.yhbeh.2009.08.006

52. Ben Ammar I, Baeklandt S, Cornet V, Antipine S, Sonny D, Mandiki SNM, et al. Passage through a hydropower plant affects the physiological and health status of Atlantic salmon smolts. Comp Biochem Physiol A Mol Integr Physiol. 2020;247: 110745. doi:10.1016/j.cbpa.2020.110745

53. Benedito-Palos L, Ballester-Lozano GF, Simó P, Karalazos V, Ortiz Á, Calduch-Giner J, et al. Lasting effects of butyrate and low FM/FO diets on growth performance, blood haematology/biochemistry and molecular growth-related markers in gilthead sea bream (Sparus aurata). Aquaculture. 2016;454: 8–18. doi:10.1016/j.aquaculture.2015.12.008

54. Benedito-Palos L, Calduch-Giner JA, Ballester-Lozano GF, Perez-Sanchez J. Effect of ration size on fillet fatty acid composition, phospholipid allostasis and mRNA expression patterns of lipid regulatory genes in gilthead sea bream (Sparus aurata). Br J Nutr. 2013;109: 1175–1187. doi:10.1017/S000711451200311X

55. Benhaiem S, Hofer H, Dehnhard M, Helms J, East ML. Sibling competition and hunger increase allostatic load in spotted hyaenas. Biol Lett. 2013;9: 20130040. doi:10.1098/rsbl.2013.0040

56. Benítez-Dorta V, Caballero MJ, Betancor MB, Manchado M, Tort L, Torrecillas S, et al. Effects of thermal stress on the expression of glucocorticoid receptor complex linked genes in Senegalese sole (Solea senegalensis): Acute and adaptive stress responses. Gen Comp Endocrinol. 2017;252: 173–185. doi:10.1016/j.ygcen.2017.06.022

57. Bergamin C, Comin A, Corazzin M, Faustini M, Peric T, Scollo A, et al. Cortisol, DHEA, and sexual steroid concentrations in fattening pigs’ hair. Animals. 2019;9: 345. doi:10.3390/ani9060345

58. Bermejo-Nogales A, Calduch-Giner JA, Pérez-Sánchez J. Unraveling the Molecular Signatures of Oxidative Phosphorylation to Cope with the Nutritionally Changing Metabolic Capabilities of Liver and Muscle Tissues in Farmed Fish. PLOS ONE. 2015;10: e0122889. doi:10.1371/journal.pone.0122889

59. Bermejo-Nogales A, Nederlof M, Benedito-Palos L, Ballester-Lozano GF, Folkedal O, Olsen RE, et al. Metabolic and transcriptional responses of gilthead sea bream (Sparus aurata L.) to environmental stress: new insights in fish mitochondrial phenotyping. Gen Comp Endocrinol. 2014;205: 305–315. doi:10.1016/j.ygcen.2014.04.016

60. Biller JD, Takahashi LS, Urbinati EC. Under stress conditions, pacu Piaractus mesopotamicus modulates the metabolic allostatic load even after Dolops carvalhoi challenge to maintain self-protection mechanisms. Fish Physiol Biochem. 2020;46: 1309–1321. doi:10.1007/s10695-020-00789-6

61. Binder TR, O’Connor CM, McConnachie SH, Wilson SM, Nannini MA, Wahl DH, et al. Is winter worse for stressed fish? The consequences of exogenous cortisol manipulation on over-winter survival and condition of juvenile largemouth bass. Comp Biochem Physiol A Mol Integr Physiol. 2015;187: 97–102. doi:10.1016/j.cbpa.2015.05.008

62. Blas J, Sergio F, Winglield JC, Hiraldo F. Experimental Tests of Endocrine Function in Breeding and Nonbreeding Raptors. Physiol Biochem Zool. 2011;84: 406–416. doi:10.1086/661236

63. Blessing W, Mohammed M, Ootsuka Y. Brown adipose tissue thermogenesis, the basic rest–activity cycle, meal initiation, and bodily homeostasis in rats. Physiol Behav. 2013;121: 61–69. doi:10.1016/j.physbeh.2013.03.028

64. Blevins ZW, Wahl DH, Suski CD. Reach-Scale Land Use Drives the Stress Responses of a Resident Stream Fish. Physiol Biochem Zool. 2014;87: 113–124. doi:10.1086/670732

65. Blickley JL, Word KR, Krakauer AH, Phillips JL, Sells SN, Taff CC, et al. Experimental Chronic Noise Is Related to Elevated Fecal Corticosteroid Metabolites in Lekking Male Greater Sage-Grouse (Centrocercus urophasianus). PLOS ONE. 2012;7: e50462. doi:10.1371/journal.pone.0050462

66. Boerrigter JGJ, Bos R van den, Vis H van de, Spanings T, Flik G. Effects of density, PVC-tubes and feeding time on growth, stress and aggression in African catfish (Clarias gariepinus). Aquac Res. 2016;47: 2553–2568. doi:10.1111/are.12703

67. Boerrigter JGJ, Manuel R, Bos R van den, Roques J a. C, Spanings T, Flik G, et al. Recovery from transportation by road of farmed European eel (Anguilla anguilla). Aquac Res. 2015;46: 1248–1260. doi:10.1111/are.12284

68. Boersma GJ, Smeltzer MD, Scott KA, Scheurink AJ, Tamashiro KL, Sakai RR. Stress coping style does not determine social status, but influences the consequences of social subordination stress. Physiol Behav. 2017;178: 126–133. doi:10.1016/j.physbeh.2016.12.041

69. Boleij H, Salomons AR, Sprundel M van, Arndt SS, Ohl F. Not All Mice Are Equal: Welfare Implications of Behavioural Habituation Profiles in Four 129 Mouse Substrains. PLOS ONE. 2012;7: e42544. doi:10.1371/journal.pone.0042544

70. Bonier F. Hormones in the city: Endocrine ecology of urban birds. Horm Behav. 2012;61: 763–772. doi:10.1016/j.yhbeh.2012.03.016

71. Boonstra R. Coping with Changing Northern Environments: The Role of the Stress Axis in Birds and Mammals. Integr Comp Biol. 2004;44: 95–108. doi:10.1093/icb/44.2.95

72. Boonstra R. Reality as the leading cause of stress: rethinking the impact of chronic stress in nature. Fox C, editor. Funct Ecol. 2013;27: 11–23. doi:10.1111/1365-2435.12008

73. Boratyński JS, Jefimow M, Wojciechowski MS. Melatonin attenuates phenotypic flexibility of energy metabolism in a photoresponsive mammal, the Siberian hamster. J Exp Biol. 2017; jeb.159517. doi:10.1242/jeb.159517

74. Bourbonnais ML, Nelson TA, Cattet MRL, Darimont CT, Stenhouse GB, Janz DM. Environmental factors and habitat use influence body condition of individuals in a species at risk, the grizzly bear. Conserv Physiol. 2014;2. doi:10.1093/conphys/cou043

75. Bovenkerk B, Meijboom FLB. Fish Welfare in Aquaculture: Explicating the Chain of Interactions Between Science and Ethics. J Agric Environ Ethics. 2013;26: 41–61. doi:10.1007/s10806-012-9395-x

76. Boyers M, Parrini F, Owen-Smith N, Erasmus BFN, Hetem RS. Contrasting capabilities of two ungulate species to cope with extremes of aridity. Sci Rep. 2021;11: 4216. doi:10.1038/s41598-021-83732-w

77. Boyle WA, Norris DR, Guglielmo CG. Storms drive altitudinal migration in a tropical bird. Proc R Soc Biol Sci Ser B. 2010;277: 2511–2519. doi:10.1098/rspb.2010.0344

78. Bozinovic F, Rojas JM, Broitman BR, Vásquez RA. Basal metabolism is correlated with habitat productivity among populations of degus (Octodon degus). Comp Biochem Physiol A Mol Integr Physiol. 2009;152: 560–564. doi:10.1016/j.cbpa.2008.12.015

79. Bradshaw D. Environmental endocrinology. Gen Comp Endocrinol. 2007;152: 125–141. doi:10.1016/j.ygcen.2006.12.026

80. Bradshaw SD. A state of non-specific tension in living matter? Stress in Australian animals. Gen Comp Endocrinol. 2017;244: 118–129. doi:10.1016/j.ygcen.2015.10.002

81. Braithwaite VA, Ebbesson LOE. Pain and stress responses in farmed fish: -EN- Pain and stress responses in farmed fish -FR- La douleur et les réactions de stress chez les poissons d’élevage -ES- Respuestas al dolor y el estrés en los peces de cultivo. Rev Sci Tech OIE. 2014;33: 245–253. doi:10.20506/rst.33.1.2285

82. Brand B, Hadlich F, Brandt B, Schauer N, Graunke KL, Langbein J, et al. Temperament Type Specific Metabolite Profiles of the Prefrontal Cortex and Serum in Cattle. PLOS ONE. 2015;10: e0125044. doi:10.1371/journal.pone.0125044

83. Brent LJN, Semple S, Dubuc C, Heistermann M, MacLarnon A. Social capital and physiological stress levels in free-ranging adult female rhesus macaques. Physiol Behav. 2011;102: 76–83. doi:10.1016/j.physbeh.2010.09.022

84. Brijs J, Sandblom E, Axelsson M, Sundell K, Sundh H, Kiessling A, et al. Remote physiological monitoring provides unique insights on the cardiovascular performance and stress responses of freely swimming rainbow trout in aquaculture. Sci Rep. 2019;9: 9090. doi:10.1038/s41598-019-45657-3

85. Brijs J, Sandblom E, Rosengren M, Sundell K, Berg C, Axelsson M, et al. Prospects and pitfalls of using heart rate bio-loggers to assess the welfare of rainbow trout (Oncorhynchus mykiss) in aquaculture. Aquaculture. 2019;509: 188–197. doi:10.1016/j.aquaculture.2019.05.007

86. Brischoux F, Beaugeard E, Mohring B, Parenteau C, Angelier F. Short-term dehydration influences baseline but not stress-induced corticosterone levels in the house sparrow ( *Passer domesticus* ). J Exp Biol. 2020;223: jeb216424. doi:10.1242/jeb.216424

87. Brischoux F, Lendvai ÁZ, Bókony V, Chastel O, Angelier F. Marine lifestyle is associated with higher baseline corticosterone levels in birds: Marine Lifestyle and Corticosterone in Birds. Biol J Linn Soc. 2015;115: 154–161. doi:10.1111/bij.12493

88. Brooks EJ, Mandelman JW, Sloman KA, Liss S, Danylchuk AJ, Cooke SJ, et al. The physiological response of the Caribbean reef shark (Carcharhinus perezi) to longline capture. Comp Biochem Physiol A Mol Integr Physiol. 2012;162: 94–100. doi:10.1016/j.cbpa.2011.04.012

89. Brusch GA, DeNardo DF, Lourdais O. Reproductive state and water deprivation increase plasma corticosterone in a capital breeder. Gen Comp Endocrinol. 2020;288: 113375. doi:10.1016/j.ygcen.2019.113375

90. Bryan HM, Darimont CT, Paquet PC, Wynne-Edwards KE, Smits JEG. Stress and reproductive hormones reflect inter-specific social and nutritional conditions mediated by resource availability in a bear–salmon system. Conserv Physiol. 2014;2. doi:10.1093/conphys/cou010

91. Bshary R, Oliveira RF, Oliveira TSF, Canario AVM. Do cleaning organisms reduce the stress response of client reef fish? Front Zool. 2007;4: 21. doi:10.1186/1742-9994-4-21

92. Buehler DM, Bhola N, Daliborka Barjaktarov, Wolfgang Goymann, Ingrid Schwabl, B. Irene Tieleman, et al. Constitutive Immune Function Responds More Slowly to Handling Stress than Corticosterone in a Shorebird. Physiol Biochem Zool. 2008;81: 673–681. doi:10.1086/588591

93. Burgess EA, Brown JL, Lanyon JM. Sex, scarring, and stress: understanding seasonal costs in a cryptic marine mammal. Conserv Physiol. 2013;1. doi:10.1093/conphys/cot014

94. Calabrese S, Nilsen TO, Kolarevic J, Ebbesson LOE, Pedrosa C, Fivelstad S, et al. Stocking density limits for post-smolt Atlantic salmon (Salmo salar L.) with emphasis on production performance and welfare. Aquaculture. 2017;468: 363–370. doi:10.1016/j.aquaculture.2016.10.041

95. Calisi RM, Rizzo NO, Bentley GE. Seasonal differences in hypothalamic EGR-1 and GnIH expression following capture-handling stress in house sparrows (Passer domesticus). Gen Comp Endocrinol. 2008;157: 283–287. doi:10.1016/j.ygcen.2008.05.010

96. Calisi RM. An integrative overview of the role of gonadotropin-inhibitory hormone in behavior: Applying Tinbergen’s four questions. Gen Comp Endocrinol. 2014;203: 95–105. doi:10.1016/j.ygcen.2014.03.028

97. Carbonara P, Alfonso S, Zupa W, Manfrin A, Fiocchi E, Pretto T, et al. Behavioral and physiological responses to stocking density in sea bream (Sparus aurata): Do coping styles matter? Physiol Behav. 2019;212: 112698. doi:10.1016/j.physbeh.2019.112698

98. Cardoso SC, Paitio JR, Oliveira RF, Bshary R, Soares MC. Arginine vasotocin reduces levels of cooperative behaviour in a cleaner fish. Physiol Behav. 2015;139: 314–320. doi:10.1016/j.physbeh.2014.11.052

99. Cardoso SD, Teles MC, Oliveira RF. Neurogenomic mechanisms of social plasticity. J Exp Biol. 2015;218: 140–149. doi:10.1242/jeb.106997

100. Carroll G, Turner E, Dann P, Harcourt R. Prior exposure to capture heightens the corticosterone and behavioural responses of little penguins (Eudyptula minor) to acute stress. Conserv Physiol. 2016;4. doi:10.1093/conphys/cov061

101. Caslini C, Comin A, Peric T, Prandi A, Pedrotti L, Mattiello S. Use of hair cortisol analysis for comparing population status in wild red deer (Cervus elaphus) living in areas with different characteristics. Eur J Wildl Res. 2016;62: 713–723. doi:10.1007/s10344-016-1049-2

102. Castellanos-Frías E, García-Perea R, Gisbert J, Bozinovic F, Virgós E. Intraspecific variation in the energetics of the Cabrera vole. Comp Biochem Physiol A Mol Integr Physiol. 2015;190: 32–38. doi:10.1016/j.cbpa.2015.08.011

103. Chambers DL. Increased Conductivity Affects Corticosterone Levels and Prey Consumption in Larval Amphibians. J Herpetol. 2011;45: 219–223. doi:10.1670/09-211.1

104. Champagne C, Tift M, Houser D, Crocker D. Adrenal sensitivity to stress is maintained despite variation in baseline glucocorticoids in moulting seals. Conserv Physiol. 2015;3: cov004. doi:10.1093/conphys/cov004

105. Charalambous R, Narayan E. A 29-year retrospective analysis of koala rescues in New South Wales, Australia. Yue B-S, editor. PLOS ONE. 2020;15: e0239182. doi:10.1371/journal.pone.0239182

106. Charpentier MJE, Givalois L, Faurie C, Soghessa O, Simon F, Kappeler PM. Seasonal glucocorticoid production correlates with a suite of small-magnitude environmental, demographic, and physiological effects in mandrills. Am J Phys Anthropol. 2018;165: 20–33. doi:10.1002/ajpa.23329

107. Colditz IG, Hine BC. Resilience in farm animals: biology, management, breeding and implications for animal welfare. Anim Prod Sci. 2016;56: 1961. doi:10.1071/AN15297

108. Colditz IG. A consideration of physiological regulation from the perspective of Bayesian enactivism. Physiol Behav. 2020;214: 112758. doi:10.1016/j.physbeh.2019.112758

109. Colditz I. Objecthood, Agency and Mutualism in Valenced Farm Animal Environments. Animals. 2018;8: 50. doi:10.3390/ani8040050

110. Comizzoli P, Holt WV. Breakthroughs and new horizons in reproductive biology of rare and endangered animal species. Biol Reprod. 2019;101: 514–525. doi:10.1093/biolre/ioz031

111. Conde-Sieira M, Alvarez R, Lopez-Patino MA, Miguez JM, Flik G, Soengas JL. ACTH-stimulated cortisol release from head kidney of rainbow trout is modulated by glucose concentration. J Exp Biol. 2013;216: 554–567. doi:10.1242/jeb.076505

112. Corbel H, Geiger S, Groscolas R. Preparing to fledge: the adrenocortical and metabolic responses to stress in king penguin chicks. Funct Ecol. 2010;24: 82–92. doi:10.1111/j.1365-2435.2009.01619.x

113. Cornelius JM, Boswell T, Jenni-Eiermann S, Breuner CW, Ramenofsky M. Contributions of endocrinology to the migration life history of birds. Gen Comp Endocrinol. 2013;190: 47–60. doi:10.1016/j.ygcen.2013.03.027

114. Cornelius JM, Perfito N, Zann R, Breuner CW, Hahn TP. Physiological trade-offs in self-maintenance: plumage molt and stress physiology in birds. J Exp Biol. 2011;214: 2768–2777. doi:10.1242/jeb.057174

115. Cornelius JM, Zylberberg M, Breuner CW, Gleiss AC, Hahn TP. Assessing the role of reproduction and stress in the spring emergence of haematozoan parasites in birds. J Exp Biol. 2014;217: 841–849. doi:10.1242/jeb.080697

116. Cornils JS, Hoelzl F, Huber N, Zink R, Gerritsmann H, Bieber C, et al. The insensitive dormouse: reproduction skipping is not caused by chronic stress in *Glis glis*. J Exp Biol. 2018;221: jeb183558. doi:10.1242/jeb.183558

117. Costantini D, Wachter B, Melzheimer J, Czirják GÁ. Socioecological and environmental predictors of physiological stress markers in a threatened feline species. Conserv Physiol. 2017;5. doi:10.1093/conphys/cox069

118. Cote J, Meylan S, Clobert J, Voituron Y. Carotenoid-based coloration, oxidative stress and corticosterone in common lizards. J Exp Biol. 2010;213: 2116–2124. doi:10.1242/jeb.040220

119. Crater AR, Barboza PS, Forster RJ. Regulation of rumen fermentation during seasonal fluctuations in food intake of muskoxen. Comp Biochem Physiol Mol Integr Physiol. 2007;146: 233–241. doi:10.1016/j.cbpa.2006.10.019

120. Creel S, Dantzer B, Goymann W, Rubenstein DR. The ecology of stress: effects of the social environment. Boonstra R, editor. Funct Ecol. 2013;27: 66–80. doi:10.1111/j.1365-2435.2012.02029.x

121. Crespi EJ, Rissler LJ, Mattheus NM, Engbrecht K, Duncan SI, Seaborn T, et al. Geophysiology of wood frogs: landscape patterns of prevalence of disease and circulating hormone concentrations across the eastern range. Integr Comp Biol. 2015;55: 602–617. doi:10.1093/icb/icv096

122. Crespi EJ, Williams TD, Jessop TS, Delehanty B. Life history and the ecology of stress: how do glucocorticoid hormones influence life-history variation in animals? Funct Ecol. 2013;27: 93–106. doi:10.1111/1365-2435.12009

123. Crossin GT, Love OP, Cooke SJ, Williams TD. Glucocorticoid manipulations in free‐living animals: considerations of dose delivery, life‐history context and reproductive state. Grindstaff J, editor. Funct Ecol. 2016;30: 116–125. doi:10.1111/1365-2435.12482

124. Crossin GT, Phillips RA, Lattin CR, Romero LM, Williams TD. Corticosterone mediated costs of reproduction link current to future breeding. Gen Comp Endocrinol. 2013;193: 112–120. doi:10.1016/j.ygcen.2013.07.011

125. Crossin GT, Trathan PN, Phillips RA, Gorman KB, Dawson A, Sakamoto KQ, et al. Corticosterone Predicts Foraging Behavior and Parental Care in Macaroni Penguins. Am Nat. 2012;180: E31–E41. doi:10.1086/666001

126. Culbert BM, Gilmour KM, Balshine S. Stress axis regulation during social ascension in a group-living cichlid fish. Horm Behav. 2018;103: 121–128. doi:10.1016/j.yhbeh.2018.06.007

127. Dansereau G, Wey TW, Legault V, Brunet MA, Kemnitz JW, Ferrucci L, et al. Conservation of physiological dysregulation signatures of aging across primates. Aging Cell. 2019;18: e12925. doi:10.1111/acel.12925

128. Dantzer B, Fletcher QE, Boonstra R, Sheriff MJ. Measures of physiological stress: a transparent or opaque window into the status, management and conservation of species? Conserv Physiol. 2014;2. doi:10.1093/conphys/cou023

129. Davidian E, Wachter B, Heckmann I, Dehnhard M, Hofer H, Hoener OP. The interplay between social rank, physiological constraints and investment in courtship in male spotted hyenas. Funct Ecol. doi:10.1111/1365-2435.13733

130. Davies NA, Gramotnev G, McAlpine C, Seabrook L, Baxter G, Lunney D, et al. Physiological Stress in Koala Populations near the Arid Edge of Their Distribution. PLOS ONE. 2013;8: e79136. doi:10.1371/journal.pone.0079136

131. Dayger CA, LeMaster MP, Lutterschmidt DI. Physiological correlates of reproductive decisions: Relationships among body condition, reproductive status, and the hypothalamus-pituitary-adrenal axis in a reptile. Horm Behav. 2018;100: 1–11. doi:10.1016/j.yhbeh.2018.02.004

132. de Assis VR de, Titon SCM, Barsotti AMG, Jr BT, Gomes FR. Effects of Acute Restraint Stress, Prolonged Captivity Stress and Transdermal Corticosterone Application on Immunocompetence and Plasma Levels of Corticosterone on the Cururu Toad (Rhinella icterica). PLOS ONE. 2015;10: e0121005. doi:10.1371/journal.pone.0121005

133. de Bruijn R, Romero LM. Artificial rain and cold wind act as stressors to captive molting and non-molting European starlings (Sturnus vulgaris). Comp Biochem Physiol A Mol Integr Physiol. 2013;164: 512–519. doi:10.1016/j.cbpa.2012.12.017

134. de Bruijn R, Romero LM. Behavioral and physiological responses of wild-caught European starlings (Sturnus vulgaris) to a minor, rapid change in ambient temperature. Comp Biochem Physiol -Mol Integr Physiol. 2011;160: 260–266. doi:10.1016/j.cbpa.2011.06.011

135. de Bruijn R, Romero LM. The role of glucocorticoids in the vertebrate response to weather. Gen Comp Endocrinol. 2018;269: 11–32. doi:10.1016/j.ygcen.2018.07.007

136. Del Giudice M, Buck CL, Chaby LE, Gormally BM, Taff CC, Thawley CJ, et al. What Is Stress? A Systems Perspective. Integr Comp Biol. 2018;58: 1019–1032. doi:10.1093/icb/icy114

137. Delehanty B, Boonstra R. Impact of live trapping on stress profiles of Richardson’s ground squirrel (Spermophilus richardsonii). Gen Comp Endocrinol. 2009;160: 176–182. doi:10.1016/j.ygcen.2008.11.011

138. Delehanty B, Boonstra R. The benefits of baseline glucocorticoid measurements: Maximal cortisol production under baseline conditions revealed in male Richardon’s ground squirrels (Urocitellus richardsonii). Gen Comp Endocrinol. 2012;178: 470–476. doi:10.1016/j.ygcen.2012.07.009

139. DeRango EJ, Prager KC, Greig DJ, Hooper AW, Crocker DE. Climate variability and life history impact stress, thyroid, and immune markers in California sea lions (Zalophus californianus) during El Niño conditions. Conserv Physiol. 2019;7: 15.

140. Dickens MJ, Romero LM. A consensus endocrine profile for chronically stressed wild animals does not exist. Gen Comp Endocrinol. 2013;191: 177–189. doi:10.1016/j.ygcen.2013.06.014

141. Douglas HD, Kitaysky AS, Kitaiskaia EV, Maccormick A, Kelly A. Size of ornament is negatively correlated with baseline corticosterone in males of a socially monogamous colonial seabird. J Comp Physiol B-Biochem Syst Environ Physiol. 2009;179: 297–304. doi:10.1007/s00360-008-0312-6

142. Douxfils J, Lambert S, Mathieu C, Milla S, Mandiki SNM, Henrotte E, et al. Influence of domestication process on immune response to repeated emersion stressors in Eurasian perch (Perca fluviatilis, L.). Comp Biochem Physiol A Mol Integr Physiol. 2014;173: 52–60. doi:10.1016/j.cbpa.2014.03.012

143. Duarte RBM, Patrono E, Borges AC, Tomaz C, Ventura R, Gasbarri A, et al. High versus low fat/sugar food affects the behavioral, but not the cortisol response of marmoset monkeys in a conditioned-place-preference task. Physiol Behav. 2015;139: 442–448. doi:10.1016/j.physbeh.2014.11.065

144. Dupont SM, Grace JK, Brischoux F, Angelier F. Post-natal corticosterone exposure affects ornaments in adult male house sparrows (Passer domesticus). Gen Comp Endocrinol. 2019;276: 45–51. doi:10.1016/j.ygcen.2019.02.021

145. Dupoué A, Angelier F, Brischoux F, DeNardo DF, Trouvé C, Parenteau C, et al. Water deprivation increases maternal corticosterone levels and enhances offspring growth in the snake Vipera aspis. J Exp Biol. 2016;219: 658–667. doi:10.1242/jeb.132639

146. Dupoué A, Brischoux F, Lourdais O, Angelier F. Influence of temperature on the corticosterone stress–response: An experiment in the Children’s python (Antaresia childreni). Gen Comp Endocrinol. 2013;193: 178–184. doi:10.1016/j.ygcen.2013.08.004

147. DuRant SE, Arciniega ML, Bauer CM, Romero LM. A test of reactive scope: Reducing reactive scope causes delayed wound healing. Gen Comp Endocrinol. 2016;236: 115–120. doi:10.1016/j.ygcen.2016.07.013

148. DuRant SE, Hopkins WA, Hepp GR, Romero LM. Energetic constraints and parental care: Is corticosterone indicative of energetic costs of incubation in a precocial bird? Horm Behav. 2013;63: 385–391. doi:10.1016/j.yhbeh.2012.12.001

149. Duval C, Cassey P, Lovell PG, Mikšík I, Reynolds SJ, Spencer KA. Eggshell Appearance Does Not Signal Maternal Corticosterone Exposure in Japanese Quail: An Experimental Study with Brown-Spotted Eggs. PLOS ONE. 2013;8: e80485. doi:10.1371/journal.pone.0080485

150. Duval C, Zimmer C, Mikšík I, Cassey P, Spencer KA. Early life stress shapes female reproductive strategy through eggshell pigmentation in Japanese quail. Gen Comp Endocrinol. 2014;208: 146–153. doi:10.1016/j.ygcen.2014.08.013

151. Earley LA, Sammons SM, Mendonca MT, Johnston CJ. Physiological consequences of an altered flow regime on Alabama bass (Micropterus henshalli). J Appl Ichthyol. 2019;35: 917–923.

152. Ebensperger LA, Tapia D, Ramírez-Estrada J, León C, Soto-Gamboa M, Hayes LD. Fecal cortisol levels predict breeding but not survival of females in the short-lived rodent, Octodon degus. Gen Comp Endocrinol. 2013;186: 164–171. doi:10.1016/j.ygcen.2013.02.044

153. Ebensperger LA. Sociality, glucocorticoids and direct fitness in the communally rearing rodent, Octodon degus. Horm Behav. 2011;60: 346–352.

154. Eckardt W, Stoinski TS, Rosenbaum S, Umuhoza MR, Santymire R. Validating faecal glucocorticoid metabolite analysis in the Virunga mountain gorilla using a natural biological stressor. Conserv Physiol. 2016;4. doi:10.1093/conphys/cow029

155. Edes AN, Crews DE. Allostatic load and biological anthropology. Am J Phys Anthropol. 2017;162: 44–70. doi:10.1002/ajpa.23146

156. Edes AN, Edwards KL, Wolfe BA, Brown JL, Crews DE. Allostatic Load Indices With Cholesterol and Triglycerides Predict Disease and Mortality Risk in Zoo-Housed Western Lowland Gorillas ( *Gorilla gorilla gorilla* ). Biomark Insights. 2020;15: 117727192091458. doi:10.1177/1177271920914585

157. Edes AN, Wolfe BA, Crews DE. EVALUATING ALLOSTATIC LOAD: A NEW APPROACH TO MEASURING LONG-TERM STRESS IN WILDLIFE. J Zoo Wildl Med. 2018;49: 272–282. doi:10.1638/2016-0070.1

158. Edes AN, Wolfe BA, Crews DE. The first multi-zoo application of an allostatic load index to western lowland gorillas (Gorilla gorilla gorilla). Gen Comp Endocrinol. 2018;266: 135–149. doi:10.1016/j.ygcen.2018.05.006

159. Edes AN, Wolfe BA, Crews DE. Testing a method to improve predictions of disease and mortality risk in western lowland gorillas ( *gorilla gorilla gorilla* ) using allostatic load. Stress. 2021;24: 76–86. doi:10.1080/10253890.2020.1748003

160. Edes AN, Wolfe BA, Crews DE. Rearing history and allostatic load in adult western lowland gorillas ( *Gorilla gorilla gorilla* ) in human care: Rearing History and Allostatic Load in Gorillas. Zoo Biol. 2016;35: 167–173. doi:10.1002/zoo.21270

161. Edes AN, Wolfe BA, Crews DE. Assessing Stress in Zoo-Housed Western Lowland Gorillas (Gorilla gorilla gorilla) Using Allostatic Load. Int J Primatol. 2016;37: 241–259. doi:10.1007/s10764-016-9899-8

162. Edwards KL, Walker SL, Bodenham RF, Ritchie H, Shultz S. Associations between social behaviour and adrenal activity in female Barbary macaques: Consequences of study design. Gen Comp Endocrinol. 2013;186: 72–79. doi:10.1016/j.ygcen.2013.02.023

163. Eikenaar C, Fritzsch A, Bairlein F. Corticosterone and migratory fueling in Northern wheatears facing different barrier crossings. Gen Comp Endocrinol. 2013;186: 181–186. doi:10.1016/j.ygcen.2013.02.042

164. Eliasen K, Patursson EJ, McAdam BJ, Pino E, Morro B, Betancor M, et al. Liver colour scoring index, carotenoids and lipid content assessment as a proxy for lumpfish (Cyclopterus lumpus L.) health and welfare condition. Sci Rep. 2020;10: 8927. doi:10.1038/s41598-020-65535-7

165. El-Kholy MS, El-Hindawy MM, Alagawany M, El-Hack MEA, El-Sayed S a. A. Use of acetylsalicylic acid as an allostatic modulator in the diets of growing Japanese quails exposed to heat stress. J Therm Biol. 2018;74: 6–13. doi:10.1016/j.jtherbio.2018.02.011

166. Emery JA, Smullen RP, Turchini GM. Tallow in Atlantic salmon feed. Aquaculture. 2014;422–423: 98–108. doi:10.1016/j.aquaculture.2013.12.004

167. Ensminger DC, Somo DA, Houser DS, Crocker DE. Metabolic responses to adrenocorticotropic hormone (ACTH) vary with life-history stage in adult male northern elephant seals. Gen Comp Endocrinol. 2014;204: 150–157. doi:10.1016/j.ygcen.2014.04.024

168. Erb WM. Wildfire smoke impacts activity and energetics of wild Bornean orangutans. Sci Rep. 2018;8: 8. doi:DOI:10.1038/s41598-018-25847-1

169. Estrada-Cárdenas P, Cruz-Moreno DG, González-Ruiz R, Peregrino-Uriarte AB, Leyva-Carrillo L, Camacho-Jiménez L, et al. Combined hypoxia and high temperature affect differentially the response of antioxidant enzymes, glutathione and hydrogen peroxide in the white shrimp Litopenaeus vannamei. Comp Biochem Physiol A Mol Integr Physiol. 2021;254: 110909. doi:10.1016/j.cbpa.2021.110909

170. Fefferman NH, Romero LM. Can physiological stress alter population persistence? A model with conservation implications. Conserv Physiol. 2013;1: cot012. doi:10.1093/conphys/cot012

171. Fernandes-de-Castilho M, Pottinger TG, Volpato GL. Chronic social stress in rainbow trout: Does it promote physiological habituation? Gen Comp Endocrinol. 2008;155: 141–147. doi:10.1016/j.ygcen.2007.04.008

172. Ferrari S, Rey S, Høglund E, Øverli Ø, Chatain B, MacKenzie S, et al. Physiological responses during acute stress recovery depend on stress coping style in European sea bass, Dicentrarchus labrax. Physiol Behav. 2020;216: 112801. doi:10.1016/j.physbeh.2020.112801

173. Finn KR, Crutchfield JP, Bliss-Moreau E. Macaques preferentially attend to visual patterns with higher fractal dimension contours. Sci Rep. 2019;9. doi:10.1038/s41598-019-46799-0

174. Firmino JP, Fernández-Alacid L, Vallejos-Vidal E, Salomón R, Sanahuja I, Tort L, et al. Carvacrol, Thymol, and Garlic Essential Oil Promote Skin Innate Immunity in Gilthead Seabream (Sparus aurata) Through the Multifactorial Modulation of the Secretory Pathway and Enhancement of Mucus Protective Capacity. Front Immunol. 2021;12: 633621. doi:10.3389/fimmu.2021.633621

175. Fischer CP, Romero LM. Chronic captivity stress in wild animals is highly species-specific. Conserv Physiol. 2018;7: 38.

176. Fitze PS, Cote J, San-Jose LM, Meylan S, Isaksson C, Andersson S, et al. Carotenoid-Based Colours Reflect the Stress Response in the Common Lizard. PLOS ONE. 2009;4: e5111. doi:10.1371/journal.pone.0005111

177. Fletcher K, Xiong Y, Fletcher E, Gustafsson L. Glucocorticoid response to both predictable and unpredictable challenges detected as corticosterone metabolites in collared flycatcher droppings. PLOS ONE. 2018;13: e0209289. doi:10.1371/journal.pone.0209289

178. Flik G, Klaren PHM, Van den Burg EH, Metz JR, Huising MO. CRF and stress in fish. Gen Comp Endocrinol. 2006;146: 36–44. doi:10.1016/j.ygcen.2005.11.005

179. Flores R, Penna M, Wingfield JC, Cuevas E, Vásquez RA, Quirici V. Effects of traffic noise exposure on corticosterone, glutathione and tonic immobility in chicks of a precocial bird. Cooke S, editor. Conserv Physiol. 2019;7: coz061. doi:10.1093/conphys/coz061

180. Fokidis HB. Sources of variation in plasma corticosterone and dehydroepiandrosterone in the male northern cardinal (Cardinalis cardinalis): I. Seasonal patterns and effects of stress and adrenocorticotropic hormone. Gen Comp Endocrinol. 2016;235: 192–200. doi:10.1016/j.ygcen.2016.05.024

181. Fokos S, Pavlidis M, Yiotis T, Tsalafouta A, Papandroulakis N, Dermon CR. Early life low intensity stress experience modifies acute stress effects on juvenile brain cell proliferation of European sea bass (D-Labrax). Behav Brain Res. 2017;317: 109–121. doi:10.1016/j.bbr.2016.09.026

182. Folkedal O, Stien LH, Torgersen T, Oppedal E, Olsen RE, Fosseidengen JE, et al. Food anticipatory behaviour as an indicator of stress response and recovery in Atlantic salmon post-smolt after exposure to acute temperature fluctuation. Physiol Behav. 2012;105: 350–356. doi:10.1016/j.physbeh.2011.08.008

183. Folkedal O, Torgersen T, Olsen RE, Fernö A, Nilsson J, Oppedal F, et al. Duration of effects of acute environmental changes on food anticipatory behaviour, feed intake, oxygen consumption, and cortisol release in Atlantic salmon parr. Physiol Behav. 2012;105: 283–291. doi:10.1016/j.physbeh.2011.07.015

184. Fowler MA, Williams TD. A Physiological Signature of the Cost of Reproduction Associated with Parental Care. Am Nat. 2017;190: 762–773. doi:10.1086/694123

185. Fragueira R, Verhulst S, Beaulieu M. Morph- and sex-specific effects of challenging conditions on maintenance parameters in the Gouldian finch. J Exp Biol. 2019;222: jeb196030. doi:10.1242/jeb.196030

186. Fraser TWK, Vindas MA, Fjelldal PG, Winberg S, Thörnqvist P-O, Øverli Ø, et al. Increased reactivity and monoamine dysregulation following stress in triploid Atlantic salmon (Salmo salar). Comp Biochem Physiol A Mol Integr Physiol. 2015;185: 125–131. doi:10.1016/j.cbpa.2015.04.004

187. Freire CA, Cuenca ALR, Leite RD, Prado AC, Rios LP, Stakowian N, et al. Biomarkers of homeostasis, allostasis, and allostatic overload in decapod crustaceans of distinct habitats and osmoregulatory strategies: an empirical approach. Comp Biochem Physiol A Mol Integr Physiol. 2020;248: 110750. doi:10.1016/j.cbpa.2020.110750

188. Frongia GN, Peric T, Leoni G, Satta V, Berlinguer F, Muzzeddu M, et al. Assessment of Cortisol and DHEA Concentrations in Griffon Vulture (Gyps fulvus) Feathers to Evaluate its Allostatic Load. Ann Anim Sci. 2020;20: 85–96. doi:10.2478/aoas-2019-0051

189. Fürtbauer I, Heistermann M, Schülke O, Ostner J. Low female stress hormone levels are predicted by same- or opposite-sex sociality depending on season in wild Assamese macaques. Psychoneuroendocrinology. 2014;48: 19–28. doi:10.1016/j.psyneuen.2014.05.022

190. Fusi J, Peric T, Probo M, Cotticelli A, Faustini M, Veronesi MC. How Stressful Is Maternity? Study about Cortisol and Dehydroepiandrosterone-Sulfate Coat and Claws Concentrations in Female Dogs from Mating to 60 Days Post-Partum. Animals. 2021;11: 1632. doi:10.3390/ani11061632

191. Gangloff EJ, Holden KG, Telemeco RS, Baumgard LH, Bronikowski AM. Hormonal and metabolic responses to upper temperature extremes in divergent life-history ecotypes of a garter snake. J Exp Biol. 2016;219: 2944–2954. doi:10.1242/jeb.143107

192. Gangloff EJ, Sparkman AM, Holden KG, Corwin CJ, Topf M, Bronikowski AM. Geographic variation and within-individual correlations of physiological stress markers in a widespread reptile, the common garter snake (Thamnophis sirtalis). Comp Biochem Physiol A Mol Integr Physiol. 2017;205: 68–76. doi:10.1016/j.cbpa.2016.12.019

193. Gavassa S, Stoddard PK. Food restriction promotes signaling effort in response to social challenge in a short-lived electric fish. Horm Behav. 2012;62: 381–388. doi:10.1016/j.yhbeh.2012.07.003

194. Gesto M, Madsen L, Andersen NR, Jokumsen A. Differences in stress and disease resilience related to emergence time for first feeding in farmed rainbow trout ( *Oncorhynchus mykiss* ). J Exp Biol. 2018;221: jeb174623. doi:10.1242/jeb.174623

195. Gesto M, Otero-Rodiño C, López-Patiño MA, Míguez JM, Soengas JL, Conde-Sieira M. Is plasma cortisol response to stress in rainbow trout regulated by catecholamine-induced hyperglycemia? Gen Comp Endocrinol. 2014;205: 207–217. doi:10.1016/j.ygcen.2014.04.002

196. Gil D, Alfonso-Iñiguez S, Pérez-Rodríguez L, Muriel J, Monclús R. Harsh conditions during early development influence telomere length in an altricial passerine: Links with oxidative stress and corticosteroids. J Evol Biol. 2019;32: 111–125. doi:10.1111/jeb.13396

197. Glucs ZE, Smith DR, Tubbs CW, Scherbinski JJ, Welch A, Burnett J, et al. Glucocorticoid measurement in plasma, urates, and feathers from California condors (Gymnogyps californianus) in response to a human-induced stressor. PLOS ONE. 2018;13: e0205565. doi:10.1371/journal.pone.0205565

198. Gobush KS. Validation and application of noninvasive glucocorticoid and thyroid hormone measures in free-ranging Hawaiian monk seals. Gen Comp Endocrinol. 2014;195: 174–182.

199. Goessling JM, Guyer C, Mendonça MT. Seasonal Acclimation of Constitutive Immunity in Gopher Tortoises Gopherus polyphemus. Physiol Biochem Zool. 2016;89: 487–497. doi:10.1086/688694

200. Goessling JM, Kennedy H, Mendonça MT, Wilson AE. A meta‐analysis of plasma corticosterone and heterophil : lymphocyte ratios – is there conservation of physiological stress responses over time? Grindstaff J, editor. Funct Ecol. 2015;29: 1189–1196. doi:10.1111/1365-2435.12442

201. Goessling JM, Mendonça MT. Physiological responses of gopher tortoises (Gopherus polyphemus) to trapping. Conserv Physiol. 2021;9. doi:10.1093/conphys/coab003

202. González-Gómez PL, Merrill L, Ellis VA, Venegas C, Pantoja JI, Vasquez RA, et al. Breaking down seasonality: Androgen modulation and stress response in a highly stable environment. Gen Comp Endocrinol. 2013;191: 1–12. doi:10.1016/j.ygcen.2013.05.007

203. Gormally BMG, Estrada R, Yin H, Romero LM. Recovery from repeated stressors: Physiology and behavior are affected on different timescales in house sparrows. Gen Comp Endocrinol. 2019;282: 113225. doi:10.1016/j.ygcen.2019.113225

204. Gormally BMG. Recovery periods during repeated stress impact corticosterone and behavioral responses differently in house sparrows. Horm Behav. 2019;112: 81–88.

205. Goymann W, Wingfield JC. Allostatic load, social status and stress hormones: the costs of social status matter. Anim Behav. 2004;67: 591–602. doi:10.1016/j.anbehav.2003.08.007

206. Graham SP, Freidenfelds NA, McCormick GL, Langkilde T. The impacts of invaders: Basal and acute stress glucocorticoid profiles and immune function in native lizards threatened by invasive ants. Gen Comp Endocrinol. 2012;176: 400–408. doi:10.1016/j.ygcen.2011.12.027

207. Graham SP, Freidenfelds NA, Thawley CJ, Robbins TR, Langkilde T. Are invasive species stressful? The glucocorticoid profile of native lizards exposed to invasive fire ants depends on the context. Physiol Biochem Zool. 2017;90: 328–337. doi:10.1086/689983

208. Graham SP, Kelehear C, Brown GP, Shine R. Corticosterone–immune interactions during captive stress in invading Australian cane toads (Rhinella marina). Horm Behav. 2012;62: 146–153. doi:10.1016/j.yhbeh.2012.06.001

209. Grassie C, Braithwaite VA, Nilsson J, Nilsen TO, Teien H-C, Handeland SO, et al. Aluminum exposure impacts brain plasticity and behavior in Atlantic salmon (Salmo salar). J Exp Biol. 2013;216: 3148–3155. doi:10.1242/jeb.083550

210. Green MR, McCormick CM. Sex and stress steroids in adolescence: Gonadal regulation of the hypothalamic–pituitary–adrenal axis in the rat. Gen Comp Endocrinol. 2016;234: 110–116. doi:10.1016/j.ygcen.2016.02.004

211. Greggor AL, Spencer KA, Clayton NS, Thornton A. Wild jackdaws’ reproductive success and their offspring’s stress hormones are connected to provisioning rate and brood size, not to parental neophobia. Gen Comp Endocrinol. 2017;243: 70–77. doi:10.1016/j.ygcen.2016.11.006

212. Gregorio PF, Panebianco A, Ovejero Aguilar R, Taraborelli PA, Moreno PG, Schroeder NM, et al. Linking diet quality and energy demand in free-living guanacos: an eco-physiological innovative approach. J Zool Lond. 2019;308: 243–252. doi:10.1111/jzo.12667

213. Gregório SF, Ruiz-Jarabo I, Carvalho EM, Fuentes J. Increased intestinal carbonate precipitate abundance in the sea bream (Sparus aurata L.) in response to ocean acidification. PLOS ONE. 2019;14: e0218473. doi:10.1371/journal.pone.0218473

214. Griebel IA, Fairhurst GD, Marchant TA, Clark RG. Effects of parental and nest-site characteristics on nestling quality in the Tree Swallow (Tachycineta bicolor). Can J Zool. 2019;97: 63–71. doi:10.1139/cjz-2018-0109

215. Groner ML, Rollins-Smith LA, Reinert LK, Hempel J, Bier ME, Relyea RA. Interactive effects of competition and predator cues on immune responses of leopard frogs at metamorphosis. J Exp Biol. 2014;217: 351–358. doi:doi:10.1242/jeb.091611

216. Habel J, Sundrum A. Mismatch of Glucose Allocation between Different Life Functions in the Transition Period of Dairy Cows. Animals. 2020;10: 1028. doi:10.3390/ani10061028

217. Habig B, Doellman MM, Woods K, Olansen J, Archie EA. Social status and parasitism in male and female vertebrates: a meta-analysis. Sci Rep. 2018;8: 3629. doi:10.1038/s41598-018-21994-7

218. Hämäläinen A, Heistermann M, Kraus C. The stress of growing old: sex- and season-specific effects of age on allostatic load in wild grey mouse lemurs. Oecologia. 2015;178: 1063–1075. doi:10.1007/s00442-015-3297-3

219. Hamel MJ, Spurgeon JJ, Steffensen KD, Pegg MA. Uncovering unique plasticity in life history of an endangered centenarian fish. Sci Rep. 2020;10: 12866. doi:10.1038/s41598-020-69911-1

220. Hammond TT. Contrasting stress responses of two co-occurring chipmunk species (Tamias alpinus and T. speciosus). Gen Comp Endocrinol. 2015;211: 114–122.

221. Hanson KC, Ostrand KG, Glenn RA. Physiological characterization of juvenile Chinook salmon utilizing different habitats during migration through the Columbia River Estuary. Comp Biochem Physiol A Mol Integr Physiol. 2012;163: 343–349. doi:10.1016/j.cbpa.2012.07.008

222. Harris BN, Perea-Rodriguez JP, Saltzman W. Acute effects of corticosterone injection on paternal behavior in California mouse (Peromyscus californicus) fathers. Horm Behav. 2011;60: 666–675. doi:10.1016/j.yhbeh.2011.09.001

223. Harris BN. Stress hypothesis overload: 131 hypotheses exploring the role of stress in tradeoffs, transitions, and health. Gen Comp Endocrinol. 2020;288: 113355. doi:10.1016/j.ygcen.2019.113355

224. Hayward LS, Bowles AE, Ha JC, Wasser SK. Impacts of acute and long-term vehicle exposure on physiology and reproductive success of the northern spotted owl. Ecosphere. 2011;2: art65. doi:10.1890/ES10-00199.1

225. Heimbürge S, Kanitz E, Otten W. The use of hair cortisol for the assessment of stress in animals. Gen Comp Endocrinol. 2019;270: 10–17. doi:10.1016/j.ygcen.2018.09.016

226. Heinrich SK, Hofer H, Courtiol A, Melzheimer J, Dehnhard M, Czirják GÁ, et al. Cheetahs have a stronger constitutive innate immunity than leopards. Sci Rep. 2017;7: 44837. doi:10.1038/srep44837

227. Hemmings A, Parker MO, Hale C, McBride SD. Causal and functional interpretation of mu- and delta-opioid receptor profiles in mesoaccumbens and nigrostriatal pathways of an oral stereotypy phenotype. Behav Brain Res. 2018;353: 108–113. doi:10.1016/j.bbr.2018.06.031

228. Henshaw I, Fransson T, Jakobsson S, Jenni-Eiermann S, Kullberg C. Information from the geomagnetic field triggers a reduced adrenocortical response in a migratory bird. J Exp Biol. 2009;212: 2902–2907. doi:doi:10.1242/jeb.033332

229. Hernandez SE, Strona ALS, Leiner NO, Suzán G, Romano MC. Seasonal changes of faecal cortisol metabolite levels in Gracilinanus agilis (Didelphimorphia: Didelphidae) and its association to life histories variables and parasite loads. Conserv Physiol. 2018;6: 11. doi:10.1093/conphys/coy021.

230. Hernandez-Arciga U, Herrera-Montalvo LG, Pardo MC, Valdez R, Flores-Martinez JJ, Miranda-Labra RU, et al. Stress Response in the Fishing Bat Myotis vivesi in Highly Seasonal Environments. Bat Res News. 2016;57: 66.

231. Herring G, Gawlik DE. The Role of Stress Proteins in the Study of Allostatic Overload in Birds: Use and Applicability to Current Studies in Avian Ecology. Sci World J. 2007;7: 1596–1602. doi:10.1100/tsw.2007.242

232. Hevroy EM, Tipsmark CK, Remo SC, Hansen T, Fukuda M, Torgersen T, et al. Role of the GH-IGF-1 system in Atlantic salmon and rainbow trout postsmolts at elevated water temperature. Comp Biochem Physiol Mol Integr Physiol. 2015;188: 127–138.

233. Higham JP. Field endocrinology of nonhuman primates: past, present, and future. Horm Behav. 2016;84: 145–155. doi:10.1016/j.yhbeh.2016.07.001

234. Hing S, Jones KL, Rafferty C, Thompson RCA, Narayan EJ, Godfrey SS. Wildlife in the line of fire: evaluating the stress physiology of a critically endangered Australian marsupial after bushfire. Aust J Zool. 2016;64: 385. doi:10.1071/ZO16082

235. Hing S, Narayan E, Thompson RCA, Godfrey S. A review of factors influencing the stress response in Australian marsupials. Conserv Physiol. 2014;2. doi:10.1093/conphys/cou027

236. Hjelmstedt P, Brijs J, Berg C, Axelsson M, Sandblom E, Roques J a. C, et al. Continuous physiological welfare evaluation of European whitefish (Coregonus lavaretus) during common aquaculture practices leading up to slaughter. Aquaculture. 2021;534. doi:10.1016/j.aquaculture.2020.736258

237. Hoffman CL, Higham JP, Heistermann M, Coe CL, Prendergast BJ, Maestripieri D. Immune function and HPA axis activity in free-ranging rhesus macaques. Physiol Behav. 2011;104: 507–514. doi:10.1016/j.physbeh.2011.05.021

238. Hofmann T, Schmucker SS, Bessei W, Grashorn M, Stefanski V. Impact of Housing Environment on the Immune System in Chickens: A Review. Animals. 2020;10: 1138. doi:10.3390/ani10071138

239. Hoglund E, Hogberget R, Atland A, Haraldstad T, Overli O, Vindas MA. Effects of repeated short episodes of environmental acidification on Atlantic salmon (Salmo salar) from a landlocked population. Sci Total Environ. 2021;753. doi:10.1016/j.scitotenv.2020.141403

240. Hoglund E, Korzan W, Atland A, Haraldstad T, Hogberget R, Mayer I, et al. Neuroendocrine indicators of allostatic load reveal the impact of environmental acidification in fish. Comp Biochem Physiol Part C Toxicol Pharmacol. 2020;229: 108679. doi:10.1016/j.cbpc.2019.108679

241. Holden KG, Gangloff EJ, Gomez-Mancillas E, Hagerty K, Bronikowski AM. Surviving winter: Physiological regulation of energy balance in a temperate ectotherm entering and exiting brumation. Gen Comp Endocrinol. 2021;307: 113758. doi:10.1016/j.ygcen.2021.113758

242. Homberger B, Jenni-Eiermann S, Jenni L. Distinct responses of baseline and stress-induced corticosterone levels to genetic and environmental factors. Gen Comp Endocrinol. 2015;210: 46–54. doi:10.1016/j.ygcen.2014.09.020

243. Honarmand M, Goymann W, Naguib M. Stressful dieting: nutritional conditions but not compensatory growth elevate corticosterone levels in zebra finch nestlings and fledglings. PLOS ONE. 2010;5. doi:10.1371/journal.pone.0012930

244. Hu JY, Hester PY, Xiong Y, Gates RS, Makagon MM, Cheng HW. Effect of cooled perches on the efficacy of an induced molt in White Leghorn laying hens previously exposed to heat stress. Poult Sci. 2019;98: 4290–4300. doi:10.3382/ps/pez317

245. Huber N, Fusani L, Ferretti A, Mahr K, Canoine V. Measuring short-term stress in birds: Comparing different endpoints of the endocrine-immune interface. Physiol Behav. 2017;182: 46–53. doi:10.1016/j.physbeh.2017.09.017

246. Hudson SB, Kluever BM, Webb AC, French SS. Steroid hormones, energetic state, and immunocompetence vary across reproductive contexts in a parthenogenetic lizard. Gen Comp Endocrinol. 2020;288: 113372. doi:10.1016/j.ygcen.2019.113372

247. Hudson SB, Lidgard AD, French SS. Glucocorticoids, energy metabolites, and immunity vary across allostatic states for plateau side-blotched lizards (Uta stansburiana uniformis) residing in a heterogeneous thermal environment. J Exp Zool. 2020;333. doi:10.1002/jez.2415

248. Hudson SB, Robertson MW, Wilcoxen TE. Fecal Glucocorticoid Response to Periodic Social Stress in Male Green Anoles, Anolis carolinensis. Copeia. 2019;107: 653–660. doi:10.1643/CP-19-192

249. Hundal BK, Liland NS, Rosenlund G, Höglund E, Araujo P, Stubhaug I, et al. Increasing the dietary n-6/n-3 ratio alters the hepatic eicosanoid production after acute stress in Atlantic salmon (Salmo salar). Aquaculture. 2021;534: 736272. doi:10.1016/j.aquaculture.2020.736272

250. Iacchetta MG, Maloney KN, Gienger CM. Endocrine stress response of Eastern Fence Lizards in fire-disturbed landscapes. Curr Zool. 2019;65: 643–650. doi:10.1093/cz/zoy092

251. Iversen MH, Eliassen RA. The effect of allostatic load on hypothalamic–pituitary–interrenal (HPI) axis before and after secondary vaccination in Atlantic salmon postsmolts (Salmo salar L.). Fish Physiol Biochem. 2014;40: 527–538. doi:10.1007/s10695-013-9863-x

252. Jachowski DS, Kauffman MJ, Jesmer BR, Sawyer H, Millspaugh JJ. Integrating physiological stress into the movement ecology of migratory ungulates: a spatial analysis with mule deer. Conserv Physiol. 2018;6. doi:10.1093/conphys/coy054

253. Jachowski DS, Slotow R, Millspaugh JJ. Physiological Stress and Refuge Behavior by African Elephants. PLOS ONE. 2012;7: e31818. doi:10.1371/journal.pone.0031818

254. Jankowski MD, Wittwer DJ, Heisey DM, Franson JC, Hofmeister EK. The Adrenocortical Response of Greater Sage Grouse (Centrocercusurophasianus) to Capture, ACTH Injection, and Confinement, as Measured in Fecal Samples. Physiol Biochem Zool. 2009;82: 190–201. doi:10.1086/596513

255. Jerem P, Jenni-Eiermann S, McKeegan D, McCafferty DJ, Nager RG. Eye region surface temperature dynamics during acute stress relate to baseline glucocorticoids independently of environmental conditions. Physiol Behav. 2019;210: 112627. doi:10.1016/j.physbeh.2019.112627

256. Jerez-Cepa I, Fernández-Castro M, Alameda-López M, González-Manzano G, Mancera JM, Ruiz-Jarabo I. Transport and recovery of gilthead seabream (Sparus aurata L.) sedated with AQUI-S® and etomidate: Effects on intermediary metabolism and osmoregulation. Aquaculture. 2021;530: 735745. doi:10.1016/j.aquaculture.2020.735745

257. Jerez-Cepa I, Fernandez-Castro M, Del Santo O’Neill TJ, Antonio Martos-Sitcha J, Martinez-Rodriguez G, Miguel Mancera J, et al. Transport and Recovery of Gilthead Seabream (Sparus aurata L.) Sedated With Clove Oil and MS-222: Effects on Stress Axis Regulation and Intermediary Metabolism. Front Physiol. 2019;10: 612. doi:10.3389/fphys.2019.00612

258. Jerez-Cepa I, Gorissen M, Mancera JM, Ruiz-Jarabo I. What can we learn from glucocorticoid administration in fish? Effects of cortisol and dexamethasone on intermediary metabolism of gilthead seabream (Sparus aurata L.). Comp Biochem Physiol A Mol Integr Physiol. 2019;231: 1–10. doi:10.1016/j.cbpa.2019.01.010

259. Jerez-Cepa I, Marín-Rincón A, Martínez-Rodríguez G, Ruiz-Jarabo I, Mancera JM. A natural additive in the diet to improve growth and reduce energy expenditure of gilthead seabream (Sparus aurata L.): Attenuation of high stocking density stress responses. Aquaculture. 2020;524: 735263. doi:10.1016/j.aquaculture.2020.735263

260. Jessop TS, Anson JR, Narayan E, Lockwood T. An Introduced Competitor Elevates Corticosterone Responses of a Native Lizard (Varanus varius). Physiol Biochem Zool. 2015;88: 237–245. doi:10.1086/680689

261. Jessop TS, Woodford R, Symonds MRE. Macrostress: do large-scale ecological patterns exist in the glucocorticoid stress response of vertebrates? Funct Ecol. 2013;27: 120–130. doi:10.1111/j.1365-2435.2012.02057.x

262. Jimeno B, Briga M, Hau M, Verhulst S. Male but not female zebra finches with high plasma corticosterone have lower survival. Williams T, editor. Funct Ecol. 2018;32: 713–721. doi:10.1111/1365-2435.13021

263. Jimeno B, Hau M, Verhulst S. Corticosterone levels reflect variation in metabolic rate, independent of “stress.” Sci Rep. 2018;8: 13020. doi:10.1038/s41598-018-31258-z

264. Jimeno B, Hau M, Verhulst S. Glucocorticoid–temperature association is shaped by foraging costs in individual zebra finches. J Exp Biol. 2018;221: jeb187880. doi:10.1242/jeb.187880

265. Johns DW, Marchant TA, Fairhurst GD, Speakman JR, Clark RG. Biomarker of burden: Feather corticosterone reflects energetic expenditure and allostatic overload in captive waterfowl. Williams T, editor. Funct Ecol. 2018;32: 345–357. doi:10.1111/1365-2435.12988

266. Josserand R, Dupoué A, Agostini S, Haussy C, Le Galliard J-F, Meylan S. Habitat degradation increases stress-hormone levels during the breeding season, and decreases survival and reproduction in adult common lizards. Oecologia. 2017;184: 75–86. doi:10.1007/s00442-017-3841-4

267. Kalliokoski O, Jellestad FK, Murison R. A systematic review of studies utilizing hair glucocorticoids as a measure of stress suggests the marker is more appropriate for quantifying short-term stressors. Sci Rep. 2019;9. doi:10.1038/s41598-019-48517-2

268. Kershaw JL, Hall AJ. Seasonal variation in harbour seal (Phoca vitulina) blubber cortisol - A novel indicator of physiological state? Sci Rep. 2016;6: 21889. doi:10.1038/srep21889

269. Kim DS, Chavera C, Gabor CR, Earley RL. Individual variation in ACTH-induced cortisol levels in females of a livebearing fish at different gestational stages. Gen Comp Endocrinol. 2018;261: 51–58. doi:10.1016/j.ygcen.2018.01.022

270. King GD, Chapman JM, Midwood JD, Cooke SJ, Suski CD. Watershed-Scale Land Use Activities Influence the Physiological Condition of Stream Fish. Physiol Biochem Zool. 2016;89: 10–25. doi:10.1086/684109

271. King JM, Bradshaw SD. Stress in an Island kangaroo? The Barrow Island euro, Macropus robustus isabellinus. Gen Comp Endocrinol. 2010;167: 60–67. doi:10.1016/j.ygcen.2010.02.018

272. Kolarevic J, Baeverfjord G, Takle H, Ytteborg E, Reiten BKM, Nergård S, et al. Performance and welfare of Atlantic salmon smolt reared in recirculating or flow through aquaculture systems. Aquaculture. 2014;432: 15–25. doi:10.1016/j.aquaculture.2014.03.033

273. Koolhaas JM, Bartolomucci A, Buwalda B, de Boer SF, Flügge G, Korte SM, et al. Stress revisited: A critical evaluation of the stress concept. Neurosci Biobehav Rev. 2011;35: 1291–1301. doi:10.1016/j.neubiorev.2011.02.003

274. Korte SM, De Boer SF. A robust animal model of state anxiety: fear-potentiated behaviour in the elevated plus-maze. Eur J Pharmacol. 2003;463: 163–175. doi:10.1016/S0014-2999(03)01279-2

275. Korte SM, Olivier B, Koolhaas JM. A new animal welfare concept based on allostasis. Physiol Behav. 2007;92: 422–428. doi:10.1016/j.physbeh.2006.10.018

276. Korte SM, Prins J, Vinkers CH, Olivier B. On the origin of allostasis and stress-induced pathology in farm animals: Celebrating Darwin’s legacy. Vet J. 2009;182: 378–383. doi:10.1016/j.tvjl.2009.08.023

277. Korte SM, Koolhaas JM, Wingfield JC, McEwen BS. The Darwinian concept of stress: benefits of allostasis and costs of allostatic load and the trade-offs in health and disease. Neurosci Biobehav Rev. 2005;29: 3–38. doi:10.1016/j.neubiorev.2004.08.009

278. Kostelanetz S, Dickens MJ, Romero LM. Combined effects of molt and chronic stress on heart rate, heart rate variability, and glucocorticoid physiology in European Starlings. Comp Biochem Physiol A Mol Integr Physiol. 2009;154: 493–501. doi:10.1016/j.cbpa.2009.08.005

279. Kralj-Fišer S, Scheiber IBR, Kotrschal K, Weiß BM, Wascher CAF. Glucocorticoids enhance and suppress heart rate and behaviour in time dependent manner in greylag geese (Anser anser). Physiol Behav. 2010;100: 394–400. doi:10.1016/j.physbeh.2010.04.005

280. Krause JS, Chmura HE, Pérez JH, Quach LN, Asmus A, Word KR, et al. Breeding on the leading edge of a northward range expansion: differences in morphology and the stress response in the arctic Gambel’s white-crowned sparrow. Oecologia. 2016;180: 33–44. doi:10.1007/s00442-015-3447-7

281. Krause JS, Dorsa D, Wingfield JC. Changes in plasma concentrations of progesterone, dehydroepiandrosterone and corticosterone in response to acute stress of capture, handling and restraint in two subspecies of white-crowned sparrows. Comp Biochem Physiol A Mol Integr Physiol. 2014;177: 35–40. doi:10.1016/j.cbpa.2014.07.019

282. Krause JS, Pérez JH, Meddle SL, Wingfield JC. Effects of short-term fasting on stress physiology, body condition, and locomotor activity in wintering male white-crowned sparrows. Physiol Behav. 2017;177: 282–290. doi:10.1016/j.physbeh.2017.04.026

283. Kültz D. Physiological mechanisms used by fish to cope with salinity stress. Podrabsky JE, Stillman JH, Tomanek L, editors. J Exp Biol. 2015;218: 1907–1914. doi:10.1242/jeb.118695

284. Lara RA, Vasconcelos RO. Impact of noise on development, physiological stress and behavioural patterns in larval zebrafish. Sci Rep. 2021;11: 6615. doi:10.1038/s41598-021-85296-1

285. Larsen AK, Nymo IH, Sorensen KK, Seppola M, Rodven R, Jimenez de Bagues MP, et al. Concomitant temperature stress and immune activation may increase mortality despite efficient clearance of an intracellular bacterial infection in Atlantic cod. Front Microbiol. 2018;9: 2963.

286. Lattin CR, Romero LM. Seasonal variation in glucocorticoid and mineralocorticoid receptors in metabolic tissues of the house sparrow (Passer domesticus). Gen Comp Endocrinol. 2015;214: 95–102. doi:10.1016/j.ygcen.2014.05.033

287. Laudenslager ML, Jorgensen MJ, Fairbanks LA. Developmental patterns of hair cortisol in male and female nonhuman primates: Lower hair cortisol levels in vervet males emerge at puberty. Psychoneuroendocrinology. 2012;37: 1736–1739. doi:10.1016/j.psyneuen.2012.03.015

288. Laver PN, Ganswindt A, Ganswindt SB, Alexander KA. Non-invasive monitoring of glucocorticoid metabolites in banded mongooses (Mungos mungo) in response to physiological and biological challenges. Gen Comp Endocrinol. 2012;179: 178–183. doi:10.1016/j.ygcen.2012.08.011

289. Laver PN, Ganswindt A, Ganswindt SB, Alexander KA. Effect of food limitation and reproductive activity on fecal glucocorticoid metabolite levels in banded mongooses. BMC Ecol. 2020;20: 12. doi:10.1186/s12898-020-00280-z

290. Lawrence MJ, Eliason EJ, Brownscombe JW, Gilmour KM, Mandelman JW, Gutowsky LFG, et al. Influence of supraphysiological cortisol manipulation on predator avoidance behaviors and physiological responses to a predation threat in a wild marine teleost fish. Integr Zool. 2018;13: 206–218. doi:10.1111/1749-4877.12282

291. le Roux A, Beehner JC, Bergman TJ. Female philopatry and dominance patterns in wild geladas. Am J Primatol. 2011;73: 422–430. doi:10.1002/ajp.20916

292. Leary CJ, Crocker‐Buta S. Rapid effects of elevated stress hormones on male courtship signals suggest a major role for the acute stress response in intra‐ and intersexual selection. Sockman K, editor. Funct Ecol. 2018;32: 1214–1226. doi:10.1111/1365-2435.13054

293. Legagneux P, Harms NJ, Gauthier G, Chastel O, Gilchrist HG, Bortolotti G, et al. Does Feather Corticosterone Reflect Individual Quality or External Stress in Arctic-Nesting Migratory Birds? PLOS ONE. 2013;8: e82644. doi:10.1371/journal.pone.0082644

294. Lewanzik D, Kelm DH, Greiner S, Dehnhard M, Voigt CC. Ecological correlates of cortisol levels in two bat species with contrasting feeding habits. Gen Comp Endocrinol. 2012;177: 104–112. doi:10.1016/j.ygcen.2012.02.021

295. Li D. Coping with extremes_ Remarkably blunt adrenocortical responses to acute stress in two sympatric snow finches on the Qinghai-Tibet Plateau during winter relative to other seasons. Gen Comp Endocrinol. 2020; 9.

296. Liebl AL, Shimizu T, Martin LB. Covariation among glucocorticoid regulatory elements varies seasonally in house sparrows. Gen Comp Endocrinol. 2013;183: 32–37. doi:10.1016/j.ygcen.2012.11.021

297. Lieke T, Steinberg CEW, Pan B, Perminova IV, Meinelt T, Knopf K, et al. Phenol-rich fulvic acid as a water additive enhances growth, reduces stress, and stimulates the immune system of fish in aquaculture. Sci Rep. 2021;11: 174. doi:10.1038/s41598-020-80449-0

298. Ligocki IY, Earley RL, Hellmann JK, Hamilton IM. Variation in glucocorticoid levels in relation to direct and third-party interactions in a social cichlid fish. Physiol Behav. 2015;151: 386–394. doi:10.1016/j.physbeh.2015.08.004

299. Lika K, Pavlidis M, Mitrizakis N, Samaras A, Papandroulakis N. Do experimental units of different scale affect the biological performance of European sea bass Dicentrarchus labrax larvae? J Fish Biol. 2015;86: 1271–1285. doi:10.1111/jfb.12636

300. Lima AC, Assis J, Sayanda D, Sabino J, Oliveira RF. Impact of ecotourism on the fish fauna of Bonito region (Mato Grosso do Sul State, Brazil): ecological, behavioural and physiological measures. Neotropical Ichthyol. 2014;12: 133–143. doi:10.1590/S1679-62252014000100014

301. Lind C, Moore IT, Akçay Ç, Vernasco BJ, Lorch JM, Farrell TM. Patterns of Circulating Corticosterone in a Population of Rattlesnakes Afflicted with Snake Fungal Disease: Stress Hormones as a Potential Mediator of Seasonal Cycles in Disease Severity and Outcomes. Physiol Biochem Zool. 2017;91: 765–775. doi:10.1086/695747

302. Lind M-A. Corticosterone levels correlate in wild-grown and lab-grown feathers in greenfinches (Carduelis chloris) and predict behaviour and survival in captivity. Horm Behav. 2020;118: 7.

303. Lindström KM, Hasselquist D, Wikelski M. House sparrows (Passer domesticus) adjust their social status position to their physiological costs. Horm Behav. 2005;48: 311–320. doi:10.1016/j.yhbeh.2005.04.002

304. Liss SA, Sass GG, Suski CD. Spatial and temporal influences on the physiological condition of invasive silver carp. Conserv Physiol. 2013;1. doi:10.1093/conphys/cot017

305. Lodjak J, Mägi M, Rooni U, Tilgar V. Context-dependent effects of feather corticosterone on growth rate and fledging success of wild passerine nestlings in heterogeneous habitat. Oecologia. 2015;179: 937–946. doi:10.1007/s00442-015-3357-8

306. López‐Jiménez L, Blas J, Tanferna A, Cabezas S, Marchant T, Hiraldo F, et al. Ambient temperature, body condition and sibling rivalry explain feather corticosterone levels in developing black kites. Portugal S, editor. Funct Ecol. 2016;30: 605–613. doi:10.1111/1365-2435.12539

307. López-Jiménez L, Blas J, Tanferna A, Cabezas S, Marchant T, Hiraldo F, et al. Effects of Ontogeny, Diel Rhythms, and Environmental Variation on the Adrenocortical Physiology of Semialtricial Black Kites ( *Milvus migrans* ). Physiol Biochem Zool. 2016;89: 213–224. doi:10.1086/684966

308. López-Jiménez L, Blas J, Tanferna A, Cabezas S, Marchant T, Hiraldo F, et al. Lifetime variation in feather corticosterone levels in a long-lived raptor. Oecologia. 2017;183: 315–326. doi:10.1007/s00442-016-3708-0

309. Lopez-Patino MA, Conde-Sieira M, Gesto M, Libran-Perez M, Soengas JL, Miguez JM. Melatonin partially minimizes the adverse stress effects in Senegalese sole (Solea senegalensis). Aquaculture. 2013;388/391: 165–172. doi:10.1016/j.aquaculture.2013.01.023

310. Lorenzon S, Giulianini PG, Libralato S, Martinis M, Ferrero EA. Stress effect of two different transport systems on the physiological profiles of the crab Cancer pagurus. Aquaculture. 2008;278: 156–163. doi:10.1016/j.aquaculture.2008.03.011

311. Løvmo SD, Whatmore P, Sundh H, Sigholt T, Madaro A, Bardal T, et al. Effects of Atlantic salmon (Salmo salar) fed low- and high HUFA diets on growth and midgut intestinal health. Aquaculture. 2021;539: 736653. doi:10.1016/j.aquaculture.2021.736653

312. Lucas JR, Freeberg TM, Egbert J, Schwabl H. Fecal corticosterone, body mass, and caching rates of Carolina chickadees (Poecile carolinensis) from disturbed and undisturbed sites. Horm Behav. 2006;49: 634–643. doi:10.1016/j.yhbeh.2005.12.012

313. Lucas LR, Wang C-J, McCall TJ, McEwen BS. Effects of immobilization stress on neurochemical markers in the motivational system of the male rat. Brain Res. 2007;1155: 108–115. doi:10.1016/j.brainres.2007.04.063

314. Ludwig C, Dehnhard M, Pribbenow S, Silinski-Mehr S, Hofer H, Wachter B. Asymmetric reproductive aging in cheetah (Acinonyx jubatus) females in European zoos. 2019; 7.

315. Lukowiak K, Sunada H, Teskey M, Lukowiak K, Dalesman S. Environmentally relevant stressors alter memory formation in the pond snail Lymnaea. J Exp Biol. 2014;217: 76–83. doi:10.1242/jeb.089441

316. Luo J, Siemers BM, Koselj K. How anthropogenic noise affects foraging. Glob Change Biol. 2015;21: 3278–3289. doi:10.1111/gcb.12997

317. Lutermann H, Bodenstein C, Bennett NC. Natural Parasite Infection Affects the Tolerance but Not the Response to a Simulated Secondary Parasite Infection. PLOS ONE. 2012;7: e52077. doi:10.1371/journal.pone.0052077

318. Lutterschmidt DI, Maine AR. Sex or candy? Neuroendocrine regulation of the seasonal transition from courtship to feeding behavior in male red-sided garter snakes (Thamnophis sirtalis parietalis). Horm Behav. 2014;66: 120–134. doi:10.1016/j.yhbeh.2014.01.011

319. Lynn SE. Endocrine and neuroendocrine regulation of fathering behavior in birds. Horm Behav. 2016;77: 237–248. doi:10.1016/j.yhbeh.2015.04.005

320. MacIntosh AJJ, Alados CL, Huffman MA. Fractal analysis of behaviour in a wild primate: behavioural complexity in health and disease. J R Soc Interface. 2011;8: 1497–1509. doi:10.1098/rsif.2011.0049

321. Maciuszek M, Pijanowski L, Pekala-Safinska A, Palichleb P, Błachut M, Verburg-van Kemenade BML, et al. 17α-ethinylestradiol and 4-tert-octylphenol concurrently disrupt the immune response of common carp. Fish Shellfish Immunol. 2020;107: 238–250. doi:10.1016/j.fsi.2020.10.005

322. MacLarnon AM, Sommer V, Goffe AS, Higham JP, Lodge E, Tkaczynski P, et al. Assessing adaptability and reactive scope: Introducing a new measure and illustrating its use through a case study of environmental stress in forest-living baboons. Gen Comp Endocrinol. 2015;215: 10–24. doi:10.1016/j.ygcen.2014.09.022

323. MacLeod KJ, Sheriff MJ, Ensminger DC, Owen DAS, Langkilde T. Survival and reproductive costs of repeated acute glucocorticoid elevations in a captive, wild animal. Gen Comp Endocrinol. 2018;268: 1–6. doi:10.1016/j.ygcen.2018.07.006

324. Madaro A, Fernö A, Kristiansen TS, Olsen RE, Gorissen M, Flik G, et al. Effect of predictability on the stress response to chasing in Atlantic salmon (Salmo salar L.) parr. Physiol Behav. 2016;153: 1–6. doi:10.1016/j.physbeh.2015.10.002

325. Madaro A, Olsen RE, Kristiansen TS, Ebbesson LOE, Flik G, Gorissen M. A comparative study of the response to repeated chasing stress in Atlantic salmon ( Salmo salar L.) parr and post-smolts. Comp Biochem Physiol A Mol Integr Physiol. 2016;192: 7–16. doi:10.1016/j.cbpa.2015.11.005

326. Madaro A, Olsen RE, Kristiansen TS, Ebbesson LOE, Nilsen TO, Flik G, et al. Stress in Atlantic salmon: response to unpredictable chronic stress. J Exp Biol. 2015;218: 2538–2550. doi:10.1242/jeb.120535

327. Madliger CL, Love OP. The Need for a Predictive, Context-Dependent Approach to the Application of Stress Hormones in Conservation. Conserv Biol. 2014;28: 283–287. doi:10.1111/cobi.12185

328. Maestas-Olguin CR, Parish MM, Pentkowski NS. Coyote urine, but not 2-phenylethylamine, induces a complete profile of unconditioned anti-predator defensive behaviors. Physiol Behav. 2021;229: 113210. doi:10.1016/j.physbeh.2020.113210

329. Maestripieri D, Hoffman CL. Chronic stress, allostatic load, and aging in nonhuman primates. Dev Psychopathol. 2011;23: 1187–1195. doi:10.1017/S0954579411000551

330. Maille A, Schradin C. Ecophysiology of cognition: How do environmentally induced changes in physiology affect cognitive performance? Biol Rev Camb. 2017;92: 1101–1112. doi:10.1111/brv.12270

331. Majchrzak YN, Mastromonaco GF, Korver W, Burness G. Use of salivary cortisol to evaluate the influence of rides in dromedary camels. Gen Comp Endocrinol. 2015;211: 123–130. doi:10.1016/j.ygcen.2014.11.007

332. Malandrakis EE, Dadali O, Golomazou E, Kavouras M, Dailianis S, Chadio S, et al. DNA damage and differential gene expression associated with physical stress in gilthead seabream (Sparus aurata). Gen Comp Endocrinol. 2016;236: 98–104. doi:10.1016/j.ygcen.2016.07.009

333. Malisch JL, Saltzman W, Gomes FR, Rezende EL, Jeske DR, Garland Jr. T. Baseline and Stress-Induced Plasma Corticosterone Concentrations of Mice Selectively Bred for High Voluntary Wheel Running. Physiol Biochem Zool. 2007;80: 146–156. doi:10.1086/508828

334. Maloney SK, Fuller A, Meyer LCR, Kamerman PR, Mitchell G, Mitchell D. Minimum daily core body temperature in western grey kangaroos decreases as summer advances: a seasonal pattern, or a direct response to water, heat or energy supply? J Exp Biol. 2011;214: 1813–1820. doi:10.1242/jeb.050500

335. Mandalaywala TM, Petrullo LA, Parker KJ, Maestripieri D, Higham JP. Vigilance for threat accounts for inter-individual variation in physiological responses to adversity in rhesus macaques: A cognition × environment approach. Dev Psychobiol. 2017;59: 1031–1038. doi:10.1002/dev.21572

336. Manuel R, Boerrigter J, Roques J, van der Heul J, van den Bos R, Flik G, et al. Stress in African catfish (Clarias gariepinus) following overland transportation. Fish Physiol Biochem. 2014;40: 33–44. doi:10.1007/s10695-013-9821-7

337. Manuel R, Boerrigter JGJ, Cloosterman M, Gorissen M, Flik G, van den Bos R, et al. Effects of acute stress on aggression and the cortisol response in the African sharptooth catfish Clarias gariepinus: differences between day and night. J Fish Biol. 2016;88: 2175–2187. doi:10.1111/jfb.12989

338. Manuel R, Gorissen M, Zethof J, Ebbesson LOE, van de Vis H, Flik G. Unpredictable chronic stress decreases inhibitory avoidance learning in Tuebingen long-fin zebrafish: stronger effects in the resting phase than in the active phase. J Exp Biol. 2014;217: 3919–3928. doi:doi:10.1242/jeb.109736

339. Marchant-Forde RM, Marchant-Forde JN. Pregnancy-related changes in behavior and cardiac activity in primiparous pigs. Physiol Behav. 2004;82: 815–825. doi:10.1016/j.physbeh.2004.06.021

340. Marescot L, Benhaiem S, Gimenez O, Hofer H, Lebreton J, Olarte‐Castillo XA, et al. Social status mediates the fitness costs of infection with canine distemper virus in Serengeti spotted hyenas. White C, editor. Funct Ecol. 2018;32: 1237–1250. doi:10.1111/1365-2435.13059

341. Marin MT, Cruz FC, Planeta CS. Chronic restraint or variable stresses differently affect the behavior, corticosterone secretion and body weight in rats. Physiol Behav. 2007;90: 29–35. doi:10.1016/j.physbeh.2006.08.021

342. Martin LB. Stress and immunity in wild vertebrates: Timing is everything. Gen Comp Endocrinol. 2009;163: 70–76. doi:10.1016/j.ygcen.2009.03.008

343. Martins CIM, Conceição LEC, Schrama JW. Feeding behavior and stress response explain individual differences in feed efficiency in juveniles of Nile tilapia Oreochromis niloticus. Aquaculture. 2011;312: 192–197. doi:10.1016/j.aquaculture.2010.12.035

344. Martos-Sitcha JA, Mancera JM, Calduch-Giner JA, Yúfera M, Martínez-Rodríguez G, Pérez-Sánchez J. Unraveling the Tissue-Specific Gene Signatures of Gilthead Sea Bream (Sparus aurata L.) after Hyper- and Hypo-Osmotic Challenges. PLOS ONE. 2016;11: e0148113. doi:10.1371/journal.pone.0148113

345. Martos-Sitcha JA, Wunderink YS, Straatjes J, Skrzynska AK, Mancera JM, Martínez-Rodríguez G. Different stressors induce differential responses of the CRH-stress system in the gilthead sea bream (Sparus aurata). Comp Biochem Physiol A Mol Integr Physiol. 2014;177: 49–61. doi:10.1016/j.cbpa.2014.07.021

346. Marty PR, Hodges K, Heistermann M, Agil M, Engelhardt A. Is social dispersal stressful? A study in male crested macaques (Macaca nigra). Horm Behav. 2017;87: 62–68. doi:10.1016/j.yhbeh.2016.10.018

347. Mashburn KL, Atkinson S. Variability in leptin and adrenal response in juvenile Steller sea lions (Eumetopias jubatus) to adrenocorticotropic hormone (ACTH) in different seasons. Gen Comp Endocrinol. 2008;155: 352–358. doi:10.1016/j.ygcen.2007.05.030

348. Silva de Souza Matos L, Palme R, Silva Vasconcellos A. Behavioural and hormonal effects of member replacement in captive groups of blue-fronted amazon parrots (Amazona aestiva). Behav Processes. 2017;138: 160–169. doi:10.1016/j.beproc.2017.03.006

349. McConnachie SH, Cook KV, Patterson DA, Gilmour KM, Hinch SG, Farrell AP, et al. Consequences of acute stress and cortisol manipulation on the physiology, behavior, and reproductive outcome of female Pacific salmon on spawning grounds. Horm Behav. 2012;62: 67–76. doi:10.1016/j.yhbeh.2012.05.001

350. McCormick GL, Shea K, Langkilde T. How do duration, frequency, and intensity of exogenous CORT elevation affect immune outcomes of stress? Gen Comp Endocrinol. 2015;222: 81–87. doi:10.1016/j.ygcen.2015.07.008

351. McCormick GL. Immune responses of eastern fence lizards (Sceloporus undulatus) to repeated acute elevation of corticosterone. Gen Comp Endocrinol. 2014;204: 135–140.

352. McCormley MC, Champagne CD, Deyarmin JS, Stephan AP, Crocker DE, Houser DS, et al. Repeated adrenocorticotropic hormone administration alters adrenal and thyroid hormones in free-ranging elephant seals. Conserv Physiol. 2018;6. doi:10.1093/conphys/coy040

353. McCreary JK, Erickson ZT, Paxman E, Kiss D, Montina T, Olson DM, et al. The rat cumulative allostatic load measure (rCALM): a new translational assessment of the burden of stress. Ng J, editor. Environ Epigenetics. 2019;5: dvz005. doi:10.1093/eep/dvz005

354. McEwen BS, Wingfield JC. The concept of allostasis in biology and biomedicine. Horm Behav. 2003;43: 2–15. doi:10.1016/s0018-506x(02)00024-7

355. Meillère A, Brischoux F, Parenteau C, Angelier F. Influence of Urbanization on Body Size, Condition, and Physiology in an Urban Exploiter: A Multi-Component Approach. PLOS ONE. 2015;10: e0135685. doi:10.1371/journal.pone.0135685

356. Mendonça-Furtado O, Edaes M, Palme R, Rodrigues A, Siqueira J, Izar P. Does hierarchy stability influence testosterone and cortisol levels of bearded capuchin monkeys (Sapajus libidinosus) adult males? A comparison between two wild groups. Behav Processes. 2014;109: 79–88. doi:10.1016/j.beproc.2014.09.010

357. Menzies AK, Studd EK, Majchrzak YN, Peers MJL, Boutin S, Dantzer B, et al. Body temperature, heart rate, and activity patterns of two boreal homeotherms in winter: Homeostasis, allostasis, and ecological coexistence. White C, editor. Funct Ecol. 2020;34: 2292–2301. doi:10.1111/1365-2435.13640

358. Mercier L, Palacios E, Campa-Córdova ÁI, Tovar-Ramírez D, Hernández-Herrera R, Racotta IS. Metabolic and immune responses in Pacific whiteleg shrimp Litopenaeus vannamei exposed to a repeated handling stress. Aquaculture. 2006;258: 633–640. doi:10.1016/j.aquaculture.2006.04.036

359. Mes D, van Os R, Gorissen M, Ebbesson LOE, Finstad B, Mayer I, et al. Effects of environmental enrichment on forebrain neural plasticity and survival success of stocked Atlantic salmon. J Exp Biol. 2019;222: jeb212258. doi:doi:10.1242/jeb.212258

360. Messina S, Edwards DP, Marasco V, Canoine V, Cosset CCP, Tomassi S, et al. Glucocorticoids link forest type to local abundance in tropical birds. Grindstaff J, editor. Funct Ecol. 2020;34: 1814–1825. doi:10.1111/1365-2435.13586

361. Meylan S, Haussy C, Voituron Y. Physiological actions of corticosterone and its modulation by an immune challenge in reptiles. Gen Comp Endocrinol. 2010;169: 158–166. doi:10.1016/j.ygcen.2010.08.002

362. Michopoulos V, Reding KM, Wilson ME, Toufexis D. Social subordination impairs hypothalamic–pituitary–adrenal function in female rhesus monkeys. Horm Behav. 2012;62: 389–399.

363. Midwood JD, Larsen MH, Boel M, Jepsen N, Aarestrup K, Cooke SJ. Does cortisol manipulation influence outmigration behaviour, survival and growth of sea trout? A field test of carryover effects in wild fish. Mar Ecol Prog Ser. 2014;496: 135–144. doi:10.3354/meps10524

364. Mileva VR, Fitzpatrick JL, Marsh-Rollo S, Gilmour KM, Wood CM, Balshine S. The Stress Response of the Highly Social African Cichlid Neolamprologus pulcher. Physiol Biochem Zool. 2009;82: 720–729. doi:10.1086/605937

365. Mileva VR, Gilmour KM, Balshine S. Effects of maternal stress on egg characteristics in a cooperatively breeding fish. Comp Biochem Physiol A Mol Integr Physiol. 2011;158: 22–29. doi:10.1016/j.cbpa.2010.08.017

366. Miller DA, Vleck CM, Otis DL. Individual variation in baseline and stress-induced corticosterone and prolactin levels predicts parental effort by nesting mourning doves. Horm Behav. 2009;56: 457–464. doi:10.1016/j.yhbeh.2009.08.001

367. Moltesen M, Laursen DC, Thornqvist PO, Andersson MA, Winberg S, Hoglund E. Effects of acute and chronic stress on telencephalic neurochemistry and gene expression in rainbow trout (Oncorhynchus mykiss). J Exp Biol. 2016;219: 3907–3914.

368. Monaghan P. Organismal stress, telomeres and life histories. J Exp Biol. 2014;217: 57–66. doi:10.1242/jeb.090043

369. Monclus L, Ballesteros-Cano R, Puente J de la, Lacorte S, Lopez-Bejar M. Influence of persistent organic pollutants on the endocrine stress response in free-living and captive red kites (Milvus milvus). Environ Pollut. 2018;242: 329–337.

370. Monk JE, Belson S, Lee C. Pharmacologically-induced stress has minimal impact on judgement and attention biases in sheep. Sci Rep. 2019;9: 11446. doi:10.1038/s41598-019-47691-7

371. Montillo, Caslini, Peric, Prandi, Netto, Tubaro, et al. Analysis of 19 Minerals and Cortisol in Red Deer Hair in Two Different Areas of the Stelvio National Park: A Preliminary Study. Animals. 2019;9: 492. doi:10.3390/ani9080492

372. Montillo M, Rota Nodari S, Peric T, Polloni A, Corazzin M, Bergamin C, et al. Steroids in pig hair and welfare evaluation systems: combined approaches to improve management in pig breeding? Vet Ital. 2020;56: 177–184. doi:10.12834/VetIt.1974.11885.1

373. Mora AR, Firth A, Blareau S, Vallat A, Helfenstein F. Oxidative stress affects sperm performance and ejaculate redox status in subordinate house sparrows. J Exp Biol. 2017;220: 2577–2588. doi:10.1242/jeb.154799

374. Mora AR, Meniri M, Gning O, Glauser G, Vallat A, Helfenstein F. Antioxidant allocation modulates sperm quality across changing social environments. PLOS ONE. 2017;12: e0176385. doi:10.1371/journal.pone.0176385

375. Moreira CM, Dos Santos LP, Sousa MBC, Izar P. Variation of glucocorticoid metabolite levels is associated with survival demands in immature and reproductive demands in adult wild black capuchins (Sapajus nigritus). Int J Psychol Res. 2016;9: 20–29. doi:10.21500/20112084.2303

376. Moreira M, Schrama D, Farinha AP, Cerqueira M, Raposo de Magalhães C, Carrilho R, et al. Fish Pathology Research and Diagnosis in Aquaculture of Farmed Fish; a Proteomics Perspective. Animals. 2021;11: 125. doi:10.3390/ani11010125

377. Mormède P, Andanson S, Aupérin B, Beerda B, Guémené D, Malmkvist J, et al. Exploration of the hypothalamic–pituitary–adrenal function as a tool to evaluate animal welfare. Physiol Behav. 2007;92: 317–339. doi:10.1016/j.physbeh.2006.12.003

378. Mote RS, Hill NS, Skarlupka JH, Tran VT, Walker DI, Turner ZB, et al. Toxic tall fescue grazing increases susceptibility of the Angus steer fecal microbiota and plasma/urine metabolome to environmental effects. Sci Rep. 2020;10: 2497. doi:10.1038/s41598-020-59104-1

379. Muehlenbein MP, Ancrenaz M, Sakong R, Ambu L, Prall S, Fuller G, et al. Ape Conservation Physiology: Fecal Glucocorticoid Responses in Wild Pongo pygmaeus morio following Human Visitation. PLOS ONE. 2012;7: e33357. doi:10.1371/journal.pone.0033357

380. Nagy-Reis MB, Mendonca-Furtado O, Resende B. Do social factors related to allostatic load affect stereotypy susceptibility? Management implications for captive social animals. Anim Welf. 2019;28: 183–190. doi:10.7120/09627286.28.2.183

381. Names GR, Krause JS, Schultz EM, Angelier F, Parenteau C, Ribout C, et al. Relationships between avian malaria resilience and corticosterone, testosterone and prolactin in a Hawaiian songbird. Gen Comp Endocrinol. 2021;308: 113784. doi:10.1016/j.ygcen.2021.113784

382. Narayan E. Physiological stress levels in wild koala sub-populations facing anthropogenic induced environmental trauma and disease. Sci Rep. 2019;9: 6031. doi:10.1038/s41598-019-42448-8

383. Narayan EJ. Evaluation of physiological stress in Australian wildlife: Embracing pioneering and current knowledge as a guide to future research directions. Gen Comp Endocrinol. 2017;244: 30–39. doi:10.1016/j.ygcen.2015.12.008

384. Narayan EJ. Non-invasive reproductive and stress endocrinology in amphibian conservation physiology. Conserv Physiol. 2013;1. doi:10.1093/conphys/cot011

385. Navarro JL, Lèche A, Della Costa NS, Vera Cortez M, Marin RH, Martella MB. State of the art knowledge in adrenocortical and behavioral responses to environmental challenges in a threatened South American ratite: Implications to in situ and ex-situ conservation. Gen Comp Endocrinol. 2019;273: 52–60. doi:10.1016/j.ygcen.2018.04.012

386. Neal Webb SJ, Schapiro SJ, Sherwood CC, Raghanti MA, Hopkins WD. Neutrophil to Lymphocyte Ratio (NLR) in captive chimpanzees (Pan troglodytes): The effects of sex, age, and rearing. Partida-Sanchez S, editor. PLOS ONE. 2020;15: e0244092. doi:10.1371/journal.pone.0244092

387. Nelson TC, Doukakis P, Lindley ST, Schreier AD, Hightower JE, Hildebrand LR, et al. Research Tools to Investigate Movements, Migrations, and Life History of Sturgeons (Acipenseridae), with an Emphasis on Marine-Oriented Populations. PLOS ONE. 2013;8: e71552. doi:10.1371/journal.pone.0071552

388. Nemeth M, Millesi E, Schuster D, Quint R, Wagner K-H, Wallner B. Dietary fatty acids sex-specifically modulate guinea pig postnatal development via cortisol concentrations. Sci Rep. 2018;8: 471. doi:10.1038/s41598-017-18978-4

389. Nilsson ALK, Sandell MI. Stress hormone dynamics: an adaptation to migration? Biol Lett. 2009;5: 480–483. doi:10.1098/rsbl.2009.0193

390. Nogueira SSC, Abreu SA, Peregrino H, Nogueira-Filho SLG. The Effects of Feeding Unpredictability and Classical Conditioning on Pre-Release Training of White-Lipped Peccary (Mammalia, Tayassuidae). PLOS ONE. 2014;9: e86080. doi:10.1371/journal.pone.0086080

391. Nordstad T, Moe B, Bustnes JO, Bech C, Chastel O, Goutte A, et al. Relationships between POPs and baseline corticosterone levels in black-legged kittiwakes (Rissa tridactyla) across their breeding cycle. Environ Pollut. 2012;164: 219–226. doi:10.1016/j.envpol.2012.01.044

392. Novak MA, Hamel AF, Kelly BJ, Dettmer AM, Meyer JS. Stress, the HPA axis, and nonhuman primate well-being: a review. Appl Anim Behav Sci. 2012;143: 135–149.

393. O’Connor CM, Rodela TM, Mileva VR, Balshine S, Gilmour KM. Corticosteroid receptor gene expression is related to sex and social behaviour in a social fish. Comp Biochem Physiol A Mol Integr Physiol. 2013;164: 438–446. doi:10.1016/j.cbpa.2012.12.003

394. Ode M, Asaba A, Miyazawa E, Mogi K, Kikusui T, Izawa E-I. Sex-reversed correlation between stress levels and dominance rank in a captive non-breeder flock of crows. Horm Behav. 2015;73: 131–134. doi:10.1016/j.yhbeh.2015.07.012

395. Ogi A, Mariti C, Pirrone F, Baragli P, Gazzano A. The Influence of Oxytocin on Maternal Care in Lactating Dogs. Animals. 2021;11: 1130. doi:10.3390/ani11041130

396. Okada S, Hori N, Kimoto K, Onozuka M, Sato S, Sasaguri K. Effects of biting on elevation of blood pressure and other physiological responses to stress in rats: Biting may reduce allostatic load. Brain Res. 2007;1185: 189–194. doi:10.1016/j.brainres.2007.09.030

397. Onyango PO, Gesquiere LR, Wango EO, Alberts SC, Altmann J. Persistence of maternal effects in baboons: Mother’s dominance rank at son’s conception predicts stress hormone levels in subadult males. Horm Behav. 2008;54: 319–324. doi:10.1016/j.yhbeh.2008.03.002

398. Ordóñez-Grande B, Guerreiro PM, Sanahuja I, Fernández-Alacid L, Ibarz A. Environmental Salinity Modifies Mucus Exudation and Energy Use in European Sea Bass Juveniles. Animals. 2021;11: 1580. doi:10.3390/ani11061580

399. Orr AL, Lohse LA, Drew KL, Hermes-Lima M. Physiological oxidative stress after arousal from hibernation in Arctic ground squirrel. Comp Biochem Physiol A Mol Integr Physiol. 2009;153: 213–221. doi:10.1016/j.cbpa.2009.02.016

400. Oster M, Muráni E, Ponsuksili S, D’Eath RB, Turner SP, Evans G, et al. Hepatic expression patterns in psychosocially high-stressed pigs suggest mechanisms following allostatic principles. Physiol Behav. 2014;128: 159–165. doi:10.1016/j.physbeh.2014.02.014

401. Ouyang JQ, Hau M, Bonier F. Within seasons and among years: When are corticosterone levels repeatable? Horm Behav. 2011; 6.

402. Øverli Ø, Nordgreen J, Mejdell CM, Janczak AM, Kittilsen S, Johansen IB, et al. Ectoparasitic sea lice (Lepeophtheirus salmonis) affect behavior and brain serotonergic activity in Atlantic salmon (Salmo salar L.): Perspectives on animal welfare. Physiol Behav. 2014;132: 44–50. doi:10.1016/j.physbeh.2014.04.031

403. Owen DAS, Carter ET, Holding ML, Islam K, Moore IT. Roads are associated with a blunted stress response in a North American pit viper. Gen Comp Endocrinol. 2014;202: 87–92. doi:10.1016/j.ygcen.2014.04.020

404. Oyarzún-Salazar R, Rojas JJ, Pontigo JP, Mardones O, Muñoz JLP, Dantagnan P, et al. Long-term effects of temperatures on the physiological response of juveniles of the eurythermal sub-antarctic notothenioid Eleginops maclovinus. Aquaculture. 2021;530: 735797. doi:10.1016/j.aquaculture.2020.735797

405. Palma A, Blas J, Tella JL, Cabezas S, Marchant TA, Carrete M. Differences in adrenocortical responses between urban and rural burrowing owls: poorly-known underlying mechanisms and their implications for conservation. Conserv Physiol. 2020;8. doi:10.1093/conphys/coaa054

406. Palme R. Non-invasive measurement of glucocorticoids: Advances and problems. Physiol Behav. 2019;199: 229–243. doi:10.1016/j.physbeh.2018.11.021

407. Pavlidis M, Sundvik M, Chen Y-C, Panula P. Adaptive changes in zebrafish brain in dominant–subordinate behavioral context. Behav Brain Res. 2011;225: 529–537. doi:10.1016/j.bbr.2011.08.022

408. Pawluski J, Jego P, Henry S, Bruchet A, Palme R, Coste C, et al. Low plasma cortisol and fecal cortisol metabolite measures as indicators of compromised welfare in domestic horses (Equus caballus). PLOS ONE. 2017;12: e0182257. doi:10.1371/journal.pone.0182257

409. Payne CJ, Jessop TS, Guay P-J, Johnstone M, Feore M, Mulder RA. Population, Behavioural and Physiological Responses of an Urban Population of Black Swans to an Intense Annual Noise Event. PLOS ONE. 2012;7: e45014. doi:10.1371/journal.pone.0045014

410. Pereira RJG, Granzinolli MAM, Duarte JMB. Annual profile of fecal androgen and glucocorticoid levels in free-living male American kestrels from southern mid-latitude areas. Gen Comp Endocrinol. 2010;166: 94–103. doi:10.1016/j.ygcen.2009.12.012

411. Perez C, Granadeiro JP, Dias MP, Catry P. Sex and migratory strategy influence corticosterone levels in winter-grown feathers, with positive breeding effects in a migratory pelagic seabird. Oecologia Berl. 2016;181: 1025–1033. doi:10.1007/s00442-016-3625-2

412. Peric T, Comin A, Corazzin M, Montillo M, Canavese F, Stebel M, et al. Hair cortisol concentrations in New Zealand white rabbits subjected to surgery. Anim Welf. 2018;27: 13–20. doi:10.7120/09627286.27.1.013

413. Peric T, Comin A, Corazzin M, Montillo M, Cappa A, Campanile G, et al. Hair cortisol concentrations in Holstein-Friesian and crossbreed F1 heifers. J Dairy Sci. 2013;96: 3023–3027. doi:10.3168/jds.2012-6151

414. Peric T, Corazzin M, Romanzin A, Bovolenta S, Prandi A, Montillo M, et al. Cortisol and DHEA concentrations in the hair of dairy cows managed indoor or on pasture. Livest Sci. 2017;202: 39–43. doi:10.1016/j.livsci.2017.05.020

415. Peron G, Ferrand Y, Gossmann F, Bastat C, Guenezan M, Gimenez O. Escape migration decisions in Eurasian Woodcocks: insights from survival analyses using large-scale recovery data. Behav Ecol Sociobiol. 2011;65: 1949–1955. doi:10.1007/s00265-011-1204-4

416. Petrullo LA, Mandalaywala TM, Parker KJ, Maestripieri D, Higham JP. Effects of early life adversity on cortisol/salivary alpha-amylase symmetry in free-ranging juvenile rhesus macaques. Horm Behav. 2016;86: 78–84. doi:10.1016/j.yhbeh.2016.05.004

417. Piersma T. Why marathon migrants get away with high metabolic ceilings: towards an ecology of physiological restraint. J Exp Biol. 2011;214: 295–302. doi:10.1242/jeb.046748

418. Pohlin F, Brabender K, Fluch G, Stalder G, Petit T, Walzer C. Seasonal Variations in Heart Rate Variability as an Indicator of Stress in Free-Ranging Pregnant Przewalski’s Horses (E. ferus przewalskii) within the Hortobágy National Park in Hungary. Front Physiol. 2017;8: 664. doi:10.3389/fphys.2017.00664

419. Prandi A, Peric T, Corazzin M, Comin A, Colitti M. A first survey on hair cortisol of an Alpine ibex (Capra ibex ibex) population. Anim Sci Pap Rep. 2018;36: 57–74.

420. Price K, Kittridge C, Damby Z, Hayes SG, Addis EA. Relaxing life of the city? Allostatic load in yellow-bellied marmots along a rural–urban continuum. Elizabeth A, editor. Conserv Physiol. 2018;6. doi:10.1093/conphys/coy070

421. Prunet P, Øverli Ø, Douxfils J, Bernardini G, Kestemont P, Baron D. Fish welfare and genomics. Fish Physiol Biochem. 2012;38: 43–60. doi:10.1007/s10695-011-9522-z

422. Purser J, Radford AN. Acoustic Noise Induces Attention Shifts and Reduces Foraging Performance in Three-Spined Sticklebacks (Gasterosteus aculeatus). PLOS ONE. 2011;6: e17478. doi:10.1371/journal.pone.0017478

423. Pusch EA, Bentz AB, Becker DJ, Navara KJ. Behavioral phenotype predicts physiological responses to chronic stress in proactive and reactive birds. Gen Comp Endocrinol. 2018;255: 71–77. doi:10.1016/j.ygcen.2017.10.008

424. Raubenheimer D, Simpson SJ, Tait AH. Match and mismatch: conservation physiology, nutritional ecology and the timescales of biological adaptation. Philos Trans R Soc Lond B Biol Sci. 2012;367: 1628–1646. doi:10.1098/rstb.2012.0007

425. Rebolo-Ifrán N, Carrete M, Sanz-Aguilar A, Rodríguez-Martínez S, Cabezas S, Marchant TA, et al. Links between fear of humans, stress and survival support a non-random distribution of birds among urban and rural habitats. Sci Rep. 2015;5. doi:10.1038/srep13723

426. Rensel MA, Schlinger BA. 11ß hydroxysteroid dehydrogenases regulate circulating glucocorticoids but not central gene expression. Gen Comp Endocrinol. 2021;305: 113734. doi:10.1016/j.ygcen.2021.113734

427. Ricciardella LF, Bliley JM, Feth CC, Woodley SK. Acute stressors increase plasma corticosterone and decrease locomotor activity in a terrestrial salamander (Desmognathus ochrophaeus). Physiol Behav. 2010;101: 81–86. doi:10.1016/j.physbeh.2010.04.022

428. Riechert J, Chastel O, Becker PH. Regulation of Breeding Behavior: Do Energy-Demanding Periods Induce a Change in Prolactin or Corticosterone Baseline Levels in the Common Tern (Sterna hirundo)? Physiol Biochem Zool. 2014;87: 420–431. doi:10.1086/675682

429. Rimbach R, Blanc S, Zahariev A, Gatta M, Pillay N, Schradin C. Seasonal variation in energy expenditure in a rodent inhabiting a winter-rainfall desert. J Comp Physiol B. 2018;188: 877–888. doi:10.1007/s00360-018-1168-z

430. Rimbach R, Blanc S, Zahariev A, Robin J-P, Pillay N, Schradin C. Fat content of striped mice decreases during the breeding season but not during the food-restricted dry season. J Exp Biol. 2019;222: jeb208504. doi:10.1242/jeb.208504

431. Rimbach R, Jäger J, Pillay N, Schradin C. Food Availability Is the Main Driver of Seasonal Changes in Resting Metabolic Rate in African Striped Mice (Rhabdomys pumilio). Physiol Biochem Zool. 2018;91: 826–833. doi:10.1086/696828

432. Rivera DS, Lindsay CB, Oliva CA, Codocedo JF, Bozinovic F, Inestrosa NC. Effects of long-lasting social isolation and re-socialization on cognitive performance and brain activity: a longitudinal study in Octodon degus. Sci Rep. 2020;10: 18315. doi:10.1038/s41598-020-75026-4

433. Robart AR. Declining food availability, corticosterone, and migratory response in a nomadic, irruptive migrant. Horm Behav. 2019;110: 56–67.

434. Robert KA, Vleck C, Bronikowski AM. The effects of maternal corticosterone levels on offspring behavior in fast- and slow-growth garter snakes (Thamnophis elegans). Horm Behav. 2009;55: 24–32. doi:10.1016/j.yhbeh.2008.07.008

435. Robertson JK, Mastromonaco GF, Burness G. Social hierarchy reveals thermoregulatory trade-offs in response to repeated stressors. J Exp Biol. 2020;223. doi:10.1242/jeb.229047

436. Romero LM, Dickens MJ, Cyr NE. The reactive scope model — A new model integrating homeostasis, allostasis, and stress. Horm Behav. 2009;55: 375–389. doi:10.1016/j.yhbeh.2008.12.009

437. Romero LM, Fairhurst GD. Measuring corticosterone in feathers: Strengths, limitations, and suggestions for the future. Comp Biochem Physiol A Mol Integr Physiol. 2016;202: 112–122. doi:10.1016/j.cbpa.2016.05.002

438. Romero LM, Platts SH, Schoech SJ, Wada H, Crespi E, Martin LB, et al. Understanding stress in the healthy animal – potential paths for progress. Stress. 2015;18: 491–497. doi:10.3109/10253890.2015.1073255

439. Romero LM. Using the reactive scope model to understand why stress physiology predicts survival during starvation in Galapagos marine iguanas. Gen Comp Endocrinol. 2012;176: 296–299. doi:10.1016/j.ygcen.2011.11.004

440. Ros AFH, Lusa J, Meyer M, Soares M, Oliveira RF, Brossard M, et al. Does access to the bluestreak cleaner wrasse Labroides dimidiatus affect indicators of stress and health in resident reef fishes in the Red Sea? Horm Behav. 2011;59: 151–158. doi:10.1016/j.yhbeh.2010.11.006

441. Ros AFH, Vullioud P, Bruintjes R, Vallat A, Bshary R. Intra- and interspecific challenges modulate cortisol but not androgen levels in a year-round territorial damselfish. J Exp Biol. 2014;217: 1768–1774. doi:10.1242/jeb.093666

442. Rozen-Rechels D, Dupoué A, Meylan S, Qitout K, Decencière B, Agostini S, et al. Acclimation to Water Restriction Implies Different Paces for Behavioral and Physiological Responses in a Lizard Species. Physiol Biochem Zool. 2020;93: 160–174. doi:10.1086/707409

443. Rubenstein DR, Shen S. Reproductive Conflict and the Costs of Social Status in Cooperatively Breeding Vertebrates. Am Nat. 2009;173: 650–662. doi:10.1086/597606

444. Rubio-García ME, Rubio-Lozano MS, Ponce-Alquicira E, Rosario-Cortes C, Nava GM, Castañeda-Serrano MP. Improving appearance and microbiologic quality of broiler carcasses with an allostatic modulator. Poult Sci. 2015;94: 1957–1963. doi:10.3382/ps/pev144

445. Ruiz-Jarabo I, Barany A, Jerez-Cepa I, Mancera JM, Fuentes J. Intestinal response to salinity challenge in the Senegalese sole (Solea senegalensis). Comp Biochem Physiol A Mol Integr Physiol. 2017;204: 57–64. doi:10.1016/j.cbpa.2016.11.009

446. Ruiz-Jarabo I, Gregório SF, Alves A, Mancera JM, Fuentes J. Ocean acidification compromises energy management in Sparus aurata (Pisces: Teleostei). Comp Biochem Physiol A Mol Integr Physiol. 2021;256: 110911. doi:10.1016/j.cbpa.2021.110911

447. Ruiz-Jarabo I, Gregório SF, Gaetano P, Trischitta F, Fuentes J. High rates of intestinal bicarbonate secretion in seawater tilapia (Oreochromis mossambicus). Comp Biochem Physiol A Mol Integr Physiol. 2017;207: 57–64. doi:10.1016/j.cbpa.2017.02.022

448. Ruiz-Jarabo I, Herrera M, Hachero-Cruzado I, Vargas-Chacoff L, Mancera JM, Arjona FJ. Environmental salinity and osmoregulatory processes in cultured flatfish. Aquac Res. 2015;46: 10–29. doi:10.1111/are.12424

449. Rubio Lozano MS, Méndez Medina RD, Reyes Mayorga K, Rubio García ME, Ngapo TM, Mancera KF, et al. Effects of an allostatic modulator on the behavior and blood indicators of young bulls after transport. Vet México OA. 2019;5. doi:10.22201/fmvz.24486760e.2018.4.539

450. Rubio Lozano MS, Méndez Medina RD, Reyes Mayorga K, Rubio García ME, Ovando MA, Ngapo TM, et al. Effect of an allostatic modulator on stress blood indicators and meat quality of commercial young bulls in Mexico. Meat Sci. 2015;105: 63–67. doi:10.1016/j.meatsci.2015.03.012

451. Rusch TW, Sears MW, Angilletta MJ. Lizards perceived abiotic and biotic stressors independently when competing for shade in terrestrial mesocosms. Horm Behav. 2018;106: 44–51. doi:10.1016/j.yhbeh.2018.09.002

452. Ryan CP, Anderson WG, Berkvens CN, Hare JF. Maternal Gestational Cortisol and Testosterone Are Associated with Trade-Offs in Offspring Sex and Number in a Free-Living Rodent (Urocitellus richardsonii). PLOS ONE. 2014;9: e111052. doi:10.1371/journal.pone.0111052

453. Rymer TL, Pillay N, Schradin C. Resilience to Droughts in Mammals: A Conceptual Framework for Estimating Vulnerability of a Single Species. Q Rev Biol. 2016;91: 133–176. doi:10.1086/686810

454. Sadoul B, Friggens NC, Valotaire C, Labbé L, Colson V, Prunet P, et al. Physiological and behavioral flexibility to an acute CO 2 challenge, within and between genotypes in rainbow trout. Comp Biochem Physiol A Mol Integr Physiol. 2017;209: 25–33. doi:10.1016/j.cbpa.2017.04.002

455. Salaberger T, Millard M, Makarem SE, Möstl E, Grünberger V, Krametter-Frötscher R, et al. Influence of external factors on hair cortisol concentrations. Gen Comp Endocrinol. 2016;233: 73–78. doi:10.1016/j.ygcen.2016.05.005

456. Saltzman W, Ahmed S, Fahimi A, Wittwer DJ, Wegner FH. Social suppression of female reproductive maturation and infanticidal behavior in cooperatively breeding Mongolian gerbils. Horm Behav. 2006;49: 527–537. doi:10.1016/j.yhbeh.2005.11.004

457. Salze G, McLean E, Schwarz MH, Craig SR. Dietary mannan oligosaccharide enhances salinity tolerance and gut development of larval cobia. Aquaculture. 2008;274: 148–152. doi:10.1016/j.aquaculture.2007.11.008

458. Samaras A, Espírito Santo C, Papandroulakis N, Mitrizakis N, Pavlidis M, Höglund E, et al. Allostatic Load and Stress Physiology in European Seabass (Dicentrarchus labrax L.) and Gilthead Seabream (Sparus aurata L.). Front Endocrinol. 2018;9: 451. doi:10.3389/fendo.2018.00451

459. Santos GA, Schrama JW, Capelle J, Rombout JHWM, Verreth J a. J. Effects of dissolved carbon dioxide on energy metabolism and stress responses in European seabass (Dicentrarchus labrax). Aquac Res. 2013;44: 1370–1382. doi:10.1111/j.1365-2109.2012.03142.x

460. Santymire RM, Ali N, Marinari PE, Livieri TM. Using hair cortisol analysis to understand the biological factors that affect black-footed ferret (Mustela nigripes) stress physiology. Conserv Physiol. 2021;9. doi:10.1093/conphys/coab033

461. Schaefer R, Colombelli‐Négrel D. Behavioural and heart rate responses to stressors in two populations of Little Penguins that differ in levels of human disturbance and predation risk. Ibis. 2021;163: 858–874. doi:10.1111/ibi.12925

462. Scheiber IBR, Sterenborg M, Komdeur J. Stress assessment in captive greylag geese (Anser anser)1. J Anim Sci. 2015;93: 2124–2133. doi:10.2527/jas.2014-8523

463. Schmidt KL, Pradhan DS, Shah AH, Charlier TD, Chin EH, Soma KK. Neurosteroids, immunosteroids, and the Balkanization of endocrinology. Gen Comp Endocrinol. 2008;157: 266–274. doi:10.1016/j.ygcen.2008.03.025

464. Schöberl I, Wedl M, Bauer B, Day J, Möstl E, Kotrschal K. Effects of Owner–Dog Relationship and Owner Personality on Cortisol Modulation in Human–Dog Dyads. Anthrozoös. 2012;25: 199–214. doi:10.2752/175303712X13316289505422

465. Schoech SJ, Bowman R, Bridge ES, Boughton RK. Baseline and acute levels of corticosterone in Florida Scrub-Jays (Aphelocoma coerulescens): Effects of food supplementation, suburban habitat, and year. Gen Comp Endocrinol. 2007;154: 150–160. doi:10.1016/j.ygcen.2007.05.027

466. Schoech SJ, Romero LM, Moore IT, Bonier F. Constraints, concerns and considerations about the necessity of estimating free glucocorticoid concentrations for field endocrine studies. Fox C, editor. Funct Ecol. 2013;27: 1100–1106. doi:10.1111/1365-2435.12142

467. Schoepf I, Schradin C. Endocrinology of sociality: Comparisons between sociable and solitary individuals within the same population of African striped mice. Horm Behav. 2013;64: 89–94. doi:10.1016/j.yhbeh.2013.04.011

468. Schoof VAM, Jack KM, Ziegler TE. Male Response to Female Ovulation in White-Faced Capuchins (Cebus capucinus): Variation in Fecal Testosterone, Dihydrotestosterone, and Glucocorticoids. Int J Primatol. 2014;35: 643–660. doi:10.1007/s10764-013-9742-4

469. Schradin C, Pillay N, Kondratyeva A, Yuen C-H, Schoepf I, Krackow S. Basal blood glucose concentration in free-living striped mice is influenced by food availability, ambient temperature and social tactic. Biol Lett. 2015;11. doi:10.1098/rsbl.2015.0208

470. Schradin C, Raynaud J, Arrivé M, Blanc S. Leptin levels in free ranging striped mice (Rhabdomys pumilio) increase when food decreases: the ecological leptin hypothesis. Gen Comp Endocrinol. 2014;206: 139–145. doi:10.1016/j.ygcen.2014.06.024

471. Schreck CB. Stress and fish reproduction: The roles of allostasis and hormesis. Gen Comp Endocrinol. 2010;165: 549–556. doi:10.1016/j.ygcen.2009.07.004

472. Schulte PM. What is environmental stress? Insights from fish living in a variable environment. J Exp Biol. 2014;217: 23–34. doi:10.1242/jeb.089722

473. Schultner J, Kitaysky AS, Welcker J, Hatch S. Fat or lean: adjustment of endogenous energy stores to predictable and unpredictable changes in allostatic load. Boonstra R, editor. Funct Ecol. 2013;27: 45–55. doi:10.1111/j.1365-2435.2012.02058.x

474. Seeber PA, Franz M, Dehnhard M, Ganswindt A, Greenwood AD, East ML. Plains zebra (Equus quagga) adrenocortical activity increases during times of large aggregations in the Serengeti ecosystem. Horm Behav. 2018;102: 1–9. doi:10.1016/j.yhbeh.2018.04.005

475. Segner H, Sundh H, Buchmann K, Douxfils J, Sundell KS, Mathieu C, et al. Health of farmed fish: its relation to fish welfare and its utility as welfare indicator. Kiessling A, van de Vis H, Flik G, Mackenzie S, editors. Fish Physiol Biochem. 2012;38: 85–105. doi:10.1007/s10695-011-9517-9

476. Seguel M, Perez-Venegas D, Gutierrez J, Crocker DE, DeRango EJ. Parasitism Elicits a Stress Response That Allocates Resources for Immune Function in South American Fur Seals (Arctocephalus australis). Physiol Biochem Zool. 2019;92: 326–338. doi:10.1086/702960

477. Sepp T, Sild E, Hõrak P. Hematological Condition Indexes in Greenfinches: Effects of Captivity and Diurnal Variation. Physiol Biochem Zool. 2010;83: 276–282. doi:10.1086/648580

478. Setchell JM, Smith T, Wickings EJ, Knapp LA. Factors Affecting Fecal Glucocorticoid Levels in Semi-Free-Ranging Female Mandrills (Mandrillus sphinx). Am J Primatol. 2008;70: 1023–1032. doi:10.1002/ajp.20594

479. Setchell JM, Smith T, Wickings EJ, Knapp LA. Stress, social behaviour, and secondary sexual traits in a male primate. Horm Behav. 2010;58: 720–728. doi:10.1016/j.yhbeh.2010.07.004

480. Shave JR, Derocher AE, Cherry SG, Thiemann GW. Chronic stress and body condition of wolf-killed prey in Prince Albert National Park, Saskatchewan. Fuller A, editor. Conserv Physiol. 2019;7: coz037. doi:10.1093/conphys/coz037

481. Shero MR, Krotz RT, Costa DP, Avery JP, Burns JM. How do overwinter changes in body condition and hormone profiles influence W eddell seal reproductive success? Konarzewski M, editor. Funct Ecol. 2015;29: 1278–1291. doi:10.1111/1365-2435.12434

482. Shi M, Zhang Q, Li Y, Zhang W, Liao L, Cheng Y, et al. Global gene expression profile under low-temperature conditions in the brain of the grass carp (Ctenopharyngodon idellus). PLOS ONE. 2020;15: e0239730. doi:10.1371/journal.pone.0239730

483. Shini S, Shini A, Huff GR. Effects of chronic and repeated corticosterone administration in rearing chickens on physiology, the onset of lay and egg production of hens. Physiol Behav. 2009;98: 73–77. doi:10.1016/j.physbeh.2009.04.012

484. Silva AT, Midwood JD, Aarestrup K, Pottinger TG, Madsen SS, Cooke SJ. The Influence of Sex, Parasitism, and Ontogeny on the Physiological Response of European Eels (Anguilla anguilla) to an Abiotic Stressor. Physiol Biochem Zool. 2018;91: 976–986. doi:10.1086/698689

485. Simpson SD, Purser J, Radford AN. Anthropogenic noise compromises antipredator behaviour in European eels. Glob Change Biol. 2015;21: 586–593. doi:10.1111/gcb.12685

486. Skomal GB, Mandelman JW. The physiological response to anthropogenic stressors in marine elasmobranch fishes: A review with a focus on the secondary response. Comp Biochem Physiol A Mol Integr Physiol. 2012;162: 146–155. doi:10.1016/j.cbpa.2011.10.002

487. Smith AS. Social isolation affects partner-directed social behavior and cortisol during pair formation in marmosets, Callithrix geoffroyi. Physiol Behav. 2011;104: 955–961.

488. Smith B, Flavel M, Simpson B. Quantification of salivary cortisol from captive dingoes (Canis dingo) in relation to age, sex, and breeding season: implications for captive management. Aust Mammal. 2016;38: 21–28. doi:10.1071/AM15017

489. Soares MC, Cardoso SC, Grutter AS, Oliveira RF, Bshary R. Cortisol mediates cleaner wrasse switch from cooperation to cheating and tactical deception. Horm Behav. 2014;66: 346–350. doi:10.1016/j.yhbeh.2014.06.010

490. Sokolova I. Bioenergetics in environmental adaptation and stress tolerance of aquatic ectotherms: linking physiology and ecology in a multi-stressor landscape. J Exp Biol. 2021;224: jeb236802. doi:10.1242/jeb.236802

491. Sørensen C, Johansen IB, Øverli Ø. Neural plasticity and stress coping in teleost fishes. Gen Comp Endocrinol. 2013;181: 25–34. doi:10.1016/j.ygcen.2012.12.003

492. Sorenson GH, Dey CJ, Madliger CL, Love OP. Effectiveness of baseline corticosterone as a monitoring tool for fitness: a meta-analysis in seabirds. Oecologia. 2017;183: 353–365. doi:10.1007/s00442-016-3774-3

493. Spong G, Gould NP, Sahlén E, Cromsigt JPGM, Kindberg J, DePerno CS. Large-scale spatial variation of chronic stress signals in moose. PLOS ONE. 2020;15: e0225990. doi:10.1371/journal.pone.0225990

494. Stanton MA, Heintz MR, Lonsdorf EV, Santymire RM, Lipende I, Murray CM. Maternal Behavior and Physiological Stress Levels in Wild Chimpanzees (Pan troglodytes schweinfurthii). Int J Primatol. 2015;36: 473–488. doi:10.1007/s10764-015-9836-2

495. Starling AP, Charpentier MJE, Fitzpatrick C, Scordato ES, Drea CM. Seasonality, sociality, and reproduction: Long-term stressors of ring-tailed lemurs (Lemur catta). Horm Behav. 2010;57: 76–85. doi:10.1016/j.yhbeh.2009.09.016

496. Steell SC, Cooke SJ, Eliason EJ. Artificial light at night does not alter heart rate or locomotor behaviour in Caribbean spiny lobster (Panulirus argus): insights into light pollution and physiological disturbance using biologgers. Conserv Physiol. 2020;8. doi:10.1093/conphys/coaa097

497. Steinman KJ, Robeck TR. Establishing models of corticosteroid patterns during the life history of killer whales (Orcinus orca) under human care. Gen Comp Endocrinol. 2021;301: 113664. doi:10.1016/j.ygcen.2020.113664

498. Stewart HA, Noakes DLG, Cogliati KM, Peterson JT, Iversen MH, Schreck CB. Salinity effects on plasma ion levels, cortisol, and osmolality in Chinook salmon following lethal sampling. Comp Biochem Physiol A Mol Integr Physiol. 2016;192: 38–43. doi:10.1016/j.cbpa.2015.11.011

499. Subhash Peter MC. Understanding the adaptive response in vertebrates: The phenomenon of ease and ease response during post-stress acclimation. Gen Comp Endocrinol. 2013;181: 59–64. doi:10.1016/j.ygcen.2012.09.016

500. Studholme KR, Hipfner JM, Romero LM, Gormally BM, Iverson SJ, Crossin GT. Egg size is independent of variation in pre-breeding feather corticosterone in Cassin’s auklets during favorable oceanographic conditions. Gen Comp Endocrinol. 2018;268: 64–70. doi:10.1016/j.ygcen.2018.07.019

501. Sundh H, Finne-Fridell F, Ellis T, Taranger GL, Niklasson L, Pettersen EF, et al. Reduced water quality associated with higher stocking density disturbs the intestinal barrier functions of Atlantic salmon (Salmo salar L.). Aquaculture. 2019;512: 734356. doi:10.1016/j.aquaculture.2019.734356

502. Sundrum A. Metabolic Disorders in the Transition Period Indicate that the Dairy Cows’ Ability to Adapt is Overstressed. Animals. 2015;5: 978–1020. doi:10.3390/ani5040395

503. Sveen LR, Timmerhaus G, Krasnov A, Takle H, Stefansson SO, Handeland SO, et al. High fish density delays wound healing in Atlantic salmon (Salmo salar). Sci Rep. 2018;8: 16907. doi:10.1038/s41598-018-35002-5

504. Szwejser E, Verburg-van Kemenade BML, Maciuszek M, Chadzinska M. Estrogen-dependent seasonal adaptations in the immune response of fish. Horm Behav. 2017;88: 15–24. doi:10.1016/j.yhbeh.2016.10.007

505. Telemeco RS, Addis EA. Temperature has species-specific effects on corticosterone in alligator lizards. Gen Comp Endocrinol. 2014;206: 184–192. doi:10.1016/j.ygcen.2014.07.004

506. Thaker M, Lima SL, Hews DK. Acute corticosterone elevation enhances antipredator behaviors in male tree lizard morphs. Horm Behav. 2009;56: 51–57. doi:10.1016/j.yhbeh.2009.02.009

507. Thayer ZM, Wilson MA, Kim AW, Jaeggi AV. Impact of prenatal stress on offspring glucocorticoid levels: A phylogenetic meta-analysis across 14 vertebrate species. Sci Rep. 2018;8: 4942. doi:10.1038/s41598-018-23169-w

508. Thompson NA, Higham JP, Heistermann M, Vogel E, Cords M. Energy balance but not competitive environment corresponds with allostatic load during development in an Old World monkey. Horm Behav. 2020;119: 104664. doi:10.1016/j.yhbeh.2019.104664

509. Thompson NA. Understanding the links between social ties and fitness over the life cycle in primates. Behaviour. 2019;156: 859–908. doi:10.1163/1568539X-00003552

510. Tilbrook AJ, Ralph CR. Hormones, stress and the welfare of animals. Anim Prod Sci. 2018;58: 408–415. doi:10.1071/AN16808

511. Torres-Medina F, Cabezas S, Marchant TA, Wikelski M, Romero LM, Hau M, et al. Corticosterone implants produce stress-hyporesponsive birds. J Exp Biol. 2018;221: jeb173864. doi:10.1242/jeb.173864

512. Tort L. Stress and immune modulation in fish. Sunyer JO, editor. Dev Comp Immunol. 2011;35: 1366–1375. doi:10.1016/j.dci.2011.07.002

513. Trevisan C, Montillo M, Prandi A, Mkupasi EM, Ngowi HA, Johansen MV. Hair cortisol and dehydroepiandrosterone concentrations in naturally Taenia solium infected pigs in Tanzania. Gen Comp Endocrinol. 2017;246: 23–28. doi:10.1016/j.ygcen.2017.03.007

514. Tsalafouta A, Papandroulakis N, Pavlidis M. Early life stress and effects at subsequent stages of development in European sea bass (D. labrax). Aquaculture. 2015;436: 27–33. doi:10.1016/j.aquaculture.2014.10.042

515. Tschirren L, Bachmann D, Güler AC, Blaser O, Rhyner N, Seitz A, et al. MyFishCheck: A Model to Assess Fish Welfare in Aquaculture. Animals. 2021;11: 145. doi:10.3390/ani11010145

516. Tung J, Archie EA, Altmann J, Alberts SC. Cumulative early life adversity predicts longevity in wild baboons. Nat Commun. 2016;7: 11181. doi:10.1038/ncomms11181

517. Tyack PL. Implications for marine mammals of large-scale changes in the marine acoustic environment. O’Shea TJ, Odell DK, editors. J Mammal. 2008;89: 549–558. doi:10.1644/07-MAMM-S-307R.1

518. Vágási CI, Pătraș L, Pap PL, Vincze O, Mureșan C, Németh J, et al. Experimental increase in baseline corticosterone level reduces oxidative damage and enhances innate immune response. PLOS ONE. 2018;13: e0192701. doi:10.1371/journal.pone.0192701

519. Vanderzwalmen M, Edmonds E, Carey P, Snellgrove D, Sloman KA. Effect of a water conditioner on ornamental fish behaviour during commercial transport. Aquaculture. 2020;514: 734486. doi:10.1016/j.aquaculture.2019.734486

520. Vargas R, Balasch JC, Brandts I, Reyes-López F, Tort L, Teles M. Variations in the immune and metabolic response of proactive and reactive Sparus aurata under stimulation with Vibrio anguillarum vaccine. Sci Rep. 2018;8: 17352. doi:10.1038/s41598-018-35863-w

521. Vargas-Chacoff L, Moneva F, Oyarzun R, Martinez D, Munoz JLP, Bertran C, et al. Environmental salinity-modified osmoregulatory response in the sub-Antarctic notothenioid fish Eleginops maclovinus. Polar Biol. 2014;37: 1235–1245. doi:10.1007/s00300-014-1515-9

522. Varsamos S, Flik G, Pepin JF, Bonga SEW, Breuil G. Husbandry stress during early life stages affects the stress response and health status of juvenile sea bass, Dicentrarchus labrax. Fish Shellfish Immunol. 2006;20: 83–96. doi:10.1016/j.fsi.2005.04.005

523. Vázquez DE, Ilina N, Pagano EA, Zavala JA, Farina WM. Glyphosate affects the larval development of honey bees depending on the susceptibility of colonies. Reddy GVP, editor. PLOS ONE. 2018;13: e0205074. doi:10.1371/journal.pone.0205074

524. Veissier I, Boissy A. Stress and welfare: Two complementary concepts that are intrinsically related to the animal’s point of view. Physiol Behav. 2007;92: 429–433. doi:10.1016/j.physbeh.2006.11.008

525. Vera F, Antenucci CD, Zenuto RR. Different regulation of cortisol and corticosterone in the subterranean rodent Ctenomys talarum: Responses to dexamethasone, angiotensin II, potassium, and diet. Gen Comp Endocrinol. 2019;273: 108–117. doi:10.1016/j.ygcen.2018.05.019

526. Vera F, Zenuto R, Antenucci CD. Expanding the actions of cortisol and corticosterone in wild vertebrates: A necessary step to overcome the emerging challenges. Gen Comp Endocrinol. 2017;246: 337–353. doi:10.1016/j.ygcen.2017.01.010

527. Verbeek E, Colditz I, Blache D, Lee C. Chronic stress influences attentional and judgement bias and the activity of the HPA axis in sheep. Homberg J, editor. PLOS ONE. 2019;14: e0211363. doi:10.1371/journal.pone.0211363

528. Verbeek E, Oliver MH, Waas JR, McLeay LM, Blache D, Matthews LR. Reduced Cortisol and Metabolic Responses of Thin Ewes to an Acute Cold Challenge in Mid-Pregnancy: Implications for Animal Physiology and Welfare. PLOS ONE. 2012;7: e37315. doi:10.1371/journal.pone.0037315

529. Verburg-van Kemenade BML, Ribeiro CMS, Chadzinska M. Neuroendocrine–immune interaction in fish: Differential regulation of phagocyte activity by neuroendocrine factors. Gen Comp Endocrinol. 2011;172: 31–38. doi:10.1016/j.ygcen.2011.01.004

530. Verburg-van Kemenade BML, Van der Aa LM, Chadzinska M. Neuroendocrine–immune interaction: Regulation of inflammation via G-protein coupled receptors. Gen Comp Endocrinol. 2013;188: 94–101. doi:10.1016/j.ygcen.2012.11.010

531. Viblanc VA, Schull Q, Cornioley T, Stier A, Ménard J-J, Groscolas R, et al. An integrative appraisal of the hormonal and metabolic changes induced by acute stress using king penguins as a model. Gen Comp Endocrinol. 2018;269: 1–10. doi:10.1016/j.ygcen.2017.08.024

532. Vidal AC, Roldan M, Christofoletti MD, Tanaka Y, Galindo DJ, Duarte JMB. Stress in captive Blue-fronted parrots (Amazona aestiva): the animalists’ tale. Cooke S, editor. Conserv Physiol. 2019;7: coz097. doi:10.1093/conphys/coz097

533. Vindas MA, Fokos S, Pavlidis M, Hoglund E, Dionysopoulou S, Ebbesson LOE, et al. Early life stress induces long-term changes in limbic areas of a teleost fish: the role of catecholamine systems in stress coping. Sci Rep. 2018;8: 5638. doi:10.1038/s41598-018-23950-x

534. Voituron Y, Josserand R, Le Galliard J-F, Haussy C, Roussel D, Romestaing C, et al. Chronic stress, energy transduction, and free-radical production in a reptile. Oecologia. 2017;185: 195–203. doi:10.1007/s00442-017-3933-1

535. von Krogh K, Bjørndal GT, Nourizadeh-Lillabadi R, Ropstad E, Haug TM, Weltzien F-A. Cortisol differentially affects cell viability and reproduction-related gene expression in Atlantic cod pituitary cultures dependent on stage of sexual maturation. Comp Biochem Physiol A Mol Integr Physiol. 2019;236: 110517. doi:10.1016/j.cbpa.2019.06.017

536. Vuarin P, Pillay N, Schradin C. Elevated basal corticosterone levels increase disappearance risk of light but not heavy individuals in a long-term monitored rodent population. Horm Behav. 2019;113: 95–102. doi:10.1016/j.yhbeh.2019.05.001

537. Waagner D, Heckmann L-H, Malmendal A, Nielsen NChr, Holmstrup M, Bayley M. Hsp70 expression and metabolite composition in response to short-term thermal changes in Folsomia candida (Collembola). Comp Biochem Physiol A Mol Integr Physiol. 2010;157: 177–183. doi:10.1016/j.cbpa.2010.06.171

538. Wack CL, Lovern MB, Woodley SK. Transdermal delivery of corticosterone in terrestrial amphibians. Gen Comp Endocrinol. 2010;169: 269–275. doi:10.1016/j.ygcen.2010.09.004

539. Wada H, Salvante KG, Wagner E, Williams TD, Breuner CW. Ontogeny and Individual Variation in the Adrenocortical Response Zebra Finch (Taeniopygia guttata) Nestlings. Physiol Biochem Zool. 2009;82: 325–331. doi:10.1086/599320

540. Wale MA, Simpson SD, Radford AN. Noise negatively affects foraging and antipredator behaviour in shore crabs. Anim Behav. 2013;86: 111–118. doi:10.1016/j.anbehav.2013.05.001

541. Walker RH, Smith GD, Hudson SB, French SS, Walters AW. Warmer temperatures interact with salinity to weaken physiological facilitation to stress in freshwater fishes. Cooke S, editor. Conserv Physiol. 2020;8: coaa107. doi:10.1093/conphys/coaa107

542. Warburton EM, Khokhlova IS, Palme R, Surkova EN, van der Mescht L, Krasnov BR. Flea infestation, social contact, and stress in a gregarious rodent species: minimizing the potential parasitic costs of group-living. Parasitology. 2020;147: 78–86. doi:10.1017/S0031182019001185

543. Wasser SK, Azkarate JC, Booth RK, Hayward L, Hunt K, Ayres K, et al. Non-invasive measurement of thyroid hormone in feces of a diverse array of avian and mammalian species. Gen Comp Endocrinol. 2010;168: 1–7. doi:10.1016/j.ygcen.2010.04.004

544. Watts HE, Cornelius JM, Fudickar AM, Pérez J, Ramenofsky M. Understanding variation in migratory movements: A mechanistic approach. Gen Comp Endocrinol. 2018;256: 112–122. doi:10.1016/j.ygcen.2017.07.027

545. Webb LE, Veenhoven R, Harfeld JL, Jensen MB. What is animal happiness?: What is animal happiness? Ann N Y Acad Sci. 2019;1438: 62–76. doi:10.1111/nyas.13983

546. Webb MAH, Doroshov SI. Importance of environmental endocrinology in fisheries management and aquaculture of sturgeons. Gen Comp Endocrinol. 2011;170: 313–321. doi:10.1016/j.ygcen.2010.11.024

547. Wheaton CJ, Mylniczenko ND, Rimoldi JM, Hart R, O’Hara BR, Evans AN. Challenges, pitfalls and surprises: development and validation of a monoclonal antibody for enzyme immunoassay of the steroid 1a-hydroxycorticosterone in elasmobranch species. Gen Comp Endocrinol. 2018; 7.

548. Whipple AL, Ray C, Wasser M, Kitchens JN, Hove AA, Varner J, et al. Temporal vs. spatial variation in stress-associated metabolites within a population of climate-sensitive small mammals. Conserv Physiol. 2021;9. doi:10.1093/conphys/coab024

549. Whitham JC, Bryant JL, Miller LJ. Beyond Glucocorticoids: Integrating Dehydroepiandrosterone (DHEA) into Animal Welfare Research. Animals. 2020;10: 1381. doi:10.3390/ani10081381

550. Wingfield JC, Goymann W, Jalabert C, Soma KK. Concepts derived from the Challenge Hypothesis. Horm Behav. 2019;115: 104550. doi:10.1016/j.yhbeh.2019.06.014

551. Wingfield JC, Krause JS, Perez JH, Chmura HE, Nemeth Z, Word KR, et al. A mechanistic approach to understanding range shifts in a changing world: what makes a pioneer? Gen Comp Endocrinol. 2015;222: 44–53.

552. Wingfield JC, Pérez JH, Krause JS, Word KR, González-Gómez PL, Lisovski S, et al. How birds cope physiologically and behaviourally with extreme climatic events. Philos Trans R Soc B Biol Sci. 2017;372: 20160140. doi:10.1098/rstb.2016.0140

553. Wingfield JC. Control of behavioural strategies for capricious environments. Anim Behav. 2003;66: 807–816. doi:10.1006/anbe.2003.2298

554. Wingfield JC. Ecological processes and the ecology of stress: the impacts of abiotic environmental factors. Boonstra R, editor. Funct Ecol. 2013;27: 37–44. doi:10.1111/1365-2435.12039

555. Wingfield JC. Historical contributions of research on birds to behavioral neuroendocrinology. Horm Behav. 2005;48: 395–402. doi:10.1016/j.yhbeh.2005.06.003

556. Wingfield JC. The comparative biology of environmental stress: behavioural endocrinology and variation in ability to cope with novel, changing environments. Anim Behav. 2013;85: 1127–1133. doi:10.1016/j.anbehav.2013.02.018

557. Wingfield JC. THE CONCEPT OF ALLOSTASIS: COPING WITH A CAPRICIOUS ENVIRONMENT. J Mammal. 2005;86: 248–254. doi:10.1644/BHE-004.1

558. Wingfield JC. Coping with change: A framework for environmental signals and how neuroendocrine pathways might respond. Front Neuroendocrinol. 2015;37: 89–96. doi:10.1016/j.yfrne.2014.11.005

559. Wittig RM, Crockford C, Lehmann J, Whitten PL, Seyfarth RM, Cheney DL. Focused grooming networks and stress alleviation in wild female baboons. Horm Behav. 2008;54: 170–177. doi:10.1016/j.yhbeh.2008.02.009

560. Wolf TE, Bennett NC, Burroughs R, Ganswindt A. The impact of age-class and social context on fecal glucocorticoid metabolite levels in free-ranging male giraffes. Gen Comp Endocrinol. 2018;255: 26–31. doi:10.1016/j.ygcen.2017.09.022

561. Woodley SK, Lacy EL. An acute stressor alters steroid hormone levels and activity but not sexual behavior in male and female Ocoee salamanders (Desmognathus ocoee). Horm Behav. 2010;58: 427–432. doi:10.1016/j.yhbeh.2010.05.011

562. Woodruff JA, Lacey EA, Bentley GE, Kriegsfeld LJ. Effects of social environment on baseline glucocorticoid levels in a communally breeding rodent, the colonial tuco-tuco (Ctenomys sociabilis). Horm Behav. 2013;64: 566–572. doi:10.1016/j.yhbeh.2013.07.008

563. Wosnick N, Bendhack F, Leite RD, Morais RN, Freire CA. Benzocaine-induced stress in the euryhaline teleost, Centropomus parallelus and its implications for anesthesia protocols. Comp Biochem Physiol A Mol Integr Physiol. 2018;226: 32–37. doi:10.1016/j.cbpa.2018.07.021

564. Wunderink YS, de Vrieze E, Metz JR, Halm S, Martínez-Rodríguez G, Flik G, et al. Subfunctionalization of POMC paralogues in Senegalese sole (Solea senegalensis). Gen Comp Endocrinol. 2012;175: 407–415. doi:10.1016/j.ygcen.2011.11.026

565. Wunderink YS, Engels S, Halm S, Yúfera M, Martínez-Rodríguez G, Flik G, et al. Chronic and acute stress responses in Senegalese sole (Solea senegalensis): The involvement of cortisol, CRH and CRH-BP. Gen Comp Endocrinol. 2011;171: 203–210. doi:10.1016/j.ygcen.2011.01.010

566. Wunderink YS, Martínez-Rodríguez G, Yúfera M, Martín Montero I, Flik G, Mancera JM, et al. Food deprivation induces chronic stress and affects thyroid hormone metabolism in Senegalese sole (Solea senegalensis) post-larvae. Comp Biochem Physiol A Mol Integr Physiol. 2012;162: 317–322. doi:10.1016/j.cbpa.2012.03.023

567. Yao M, Denver RJ. Regulation of vertebrate corticotropin-releasing factor genes. Gen Comp Endocrinol. 2007;153: 200–216. doi:10.1016/j.ygcen.2007.01.046

568. Young AJ, Monfort SL. Stress and the costs of extra-territorial movement in a social carnivore. Biol Lett. 2009;5: 439–441. doi:10.1098/rsbl.2009.0032

569. Zebunke M, Puppe B, Langbein J. Effects of cognitive enrichment on behavioural and physiological reactions of pigs. Physiol Behav. 2013;118: 70–79. doi:10.1016/j.physbeh.2013.05.005

570. Zhang H, Chen H, Zhang Y, Li S, Lu D, Zhang H, et al. Molecular cloning, characterization and expression profiles of multiple leptin genes and a leptin receptor gene in orange-spotted grouper (Epinephelus coioides). Gen Comp Endocrinol. 2013;181: 295–305. doi:10.1016/j.ygcen.2012.09.008

571. Zhang VY, Williams CT, Palme R, Buck CL. Glucocorticoids and activity in free-living arctic ground squirrels: Interrelationships between weather, body condition, and reproduction. Horm Behav. 2020;125: 104818. doi:10.1016/j.yhbeh.2020.104818

572. Zupan M, Janczak AM, Framstad T, Zanella AJ. The effect of biting tails and having tails bitten in pigs. Physiol Behav. 2012;106: 638–644. doi:10.1016/j.physbeh.2012.04.025
