# Supplementary material for: The application of allostasis and allostatic load in animal species: A scoping review
Source: PLoS One. 2022 Aug 30;17(8):e0273838. doi: 10.1371/journal.pone.0273838 (PMC9426905; doi:10.1371/journal.pone.0273838)
Supplement: S1 Appendix — (DOCX) [file pone.0273838.s003.docx]

S2 Appendix:

Taxonomic and species breakdown of literature applying the concepts of allostasis and allostatic load to non-human species

| Taxonomic Group | Species | Number of Publications |
| --- | --- | --- |
| Invertebrates | Pacific whiteleg shrimp (*Litopenaeus vannamei*) | 2 |
|  | Caribbean spiny lobster (*Panulirus argus*)  Crab (*Cancer pagurus)*  Cricket (*Gryllus texensis*)  Freshwater prawn (*Macrobrachium acanthurus*)  Honey bee (*Apis mellifera*)  Marine crab *(Hepatus pudibundus*)  Marine/estuarine swimming crab (*Callinectes danae*)  Pond snail (*Lymnaea stagnali)*  Shore crab (*Carcinus maenas*)  Springtail (*Folsomia candida*) | 1 |
| Fish | Atlantic salmon (*Salmo salar* L.) | 21 |
|  | Gilthead seabream (*Sparus aurata L*.) | 20 |
|  | Rainbow trout (*Oncorhynchus mykiss*) | 11 |
|  | European seabass (*Dicentrarchus labax*) | 9 |
|  | Senegalese sole (*Solea senegalensis*) | 8 |
|  | Cichlid (*Neolamprologus pulcher*) | 5 |
|  | African catfish (Clarias gariepinus)  Bluestreak cleaner wrasse (*Labroides dimidiatus*)  European Eel (*Anguilla anguilla*)  Zebra fish (*Danio rerio*) | 3 |
|  | Atlantic cod (*Gadus morhua*)  Carp (Cyprinidae)  Chinook salmon (*Oncorhynchus tshawytscha*)  Largemouth bass (*Micropterus salmoides*)  Patagonian blennie (*Eleginops maclovinus*) | 2 |
|  | Alabama bass (*Micropterus henshalli*)  Atlantic killifish (*Fundulus heteroclitus*)  Atlantic wolffish (*Anarhichas lupus*)  Brown trout (*Salmo trutta*)  Caribbean reef shark (*Carcharhinus perezi*)  Chocolatedip chromis (*Chromis dimidiate*)  Lyretail anthias (*Pseudanthias squamipinnis*)  Cobia (*Rachycentron canadum*)  Common carp (*Cyprinus carpio* L.)  Creek chub (*Semotilus atromaculatus*)  Dusky Gregory (*Stegastes nigricans*)  Electric fish (*Brachyhypopomus gauderio*)  Eurasian perch (*Perca fluviatilis* L.)  European whitefish (*Coregonus lavaretus*)  Fat snook (*Centropomus parallelus*)  Flatfish (Pleuronectiformes)  Grass carp (*Ctenopharyngodon idellus*)  Green sturgeon (*Acipenser medirostris*)  Gulf sturgeon (*Acipenser oxyrinchus desotoi*)  Lined seahorse (*Hippocampus erectus*)  Lumpfish (*Cyclopterus lumpus*)  Mottled sculpin (*Cottus bairdii*)  Mountain sucker (*Catostomus platyrhynchus*)  Colorado River cutthroat trout (*Oncorhynchus clarki pleuriticus*)  Mozambique tilapia (*Oreochromis mossambicus*)  Nile tilapia (*Oreochromis niloticus*)  Orange-spotted grouper (*Epinephelus coioides*)  Pacu (*Piaractus mesopotamicus*)  Pallid sturgeon (*Scaphirhynchus albus*)  Pike cichlid (*Crenicichla lepidota*)  *Moenkhausia bonita*  Pink salmon (*Oncorhynchus gorbuscha*)  Sailfin molly (*Poecilia latipinna*)  Schoolmaster snapper (*Lutjanus apodus*)  Siberian sturgeon (*Acipenser baerii*)  Silver carp (*Hypophthalmichthys molitrix*)  Southern ray (*Hypanus americanus*)  Sturgeon and paddlefish (Clade Chondrostei) | 1 |
| Amphibian | Wood Frog (*Rana sylvatica*) | 3 |
|  | Ocoee salamander (*Desmognathus ocoee*) | 2 |
|  | Allegheny dusky salamander (*Desmognathus ochrophaeus*)  Cane toad (*Rhinella marina*)  Cururu toad (*Rhinella icterica*)  Gray treefrog (*Hyla versicolor*)  Green treefrog (*Hyla cinerea*)  Guttural toad (*Sclerophrys gutturalis*)  Jefferson salamander (*Ambystoma jeffersonianum*)  Leopard frog (*Lithobates pipiens*)  Red-legged salamander (*Plethodon shermani*) | 1 |
| Reptiles | Common lizard (*Zootoca vivipara*)  Eastern fence lizard (*Sceloporus undulatus*) | 6 |
|  | Children’s python (*Antaresia childreni)*  Degu (*Octodon degus*)  Gopher tortoise (*Gopherus polyphemus*)  Tuatara (*Sphenodon punctatus*)  Western terrestrial garter snake (*Thamnophis elegans*) | 2 |
|  | Aspic viper (*Vipera aspis*)  Checkered garter snake (*Thamnophis marcianus*)  Colorado checkered whiptail (*Aspidoscelis neotesselata)*  Common garter snake (*Thamnophis sirtalis*)  Copperhead snake (*Agkistrodon contortrix*)  Galápagos marine iguana (*Amblyrhynchus cristatus*)  Green anole (*Anolis carolinensis*)  Lace monitor (*Varanus varius*)  Mountain spiny lizard (*Sceloporus jarrovi*)  Northern alligator lizard (*Elgaria coerulea*)  Pigmy rattlesnake (*Sistrurus miliarius*)  Plateau side‐blotched lizard (*Uta stansburiana uniformis*)  Red-sided garter snake (*Thamnophis sirtalis parietalis*)  Southern alligator lizard (*Elgaria multicarinata*)  Three-spined stickleback (*Gasterosteus aculeatus*)  Tree lizard (*Urosaurus ornatus*) | 1 |
| Avian | House sparrow (*Passer domesticus*) | 15 |
|  | Zebra Finch (*Taeniopygia guttata*) | 7 |
|  | Chicken (*Gallus gallus domesticus*) | 6 |
|  | Black kite (*Milvus migrans*)  Black-legged kittiwake (*Rissa tridactyla*)  European starling (*Sturnus vulgaris*)  Japanese quail (*Coturnix japonica*) | 4 |
|  | King penguin (*Aptenodytes patagonicus*) | 3 |
|  | Black grouse (*Tetrao tetrix*)  Blue tit (*Cyanistes caeruleus*)  Blue-fronted amazon parrot (*Amazona aestiva*)  Burrowing owl (*Athene cunicularia*)  Common tern (*Sterna hirundo*)  Gambel’s white-crowned sparrow (*Zonotrichia leucophrys gambelii*)  Great tit (*Parus major*)  Greater sage-grouse (*Centrocercus urophasianus*)  Greenfinch (*Chloris chloris*)  Greylag goose (*Anser anser*)  Little penguin (*Eudyptula minor*)  Red crossbill (*Loxia curvirostra*)  Rock pigeon (*Columbia livia*)  Tree swallow (*Tachycineta bicolor*)  White crowned sparrow (*Zonotrichia leucophrys gambelii*) | 2 |
|  | American kestrel (*Falco sparverius*)  American redstart (*Setophaga ruticilla*)  Black swan (*Cygnus atratus*)  Blackbrowed albatross (*Thalassarche melanophrys*)  Black-capped babbler (*Pellorneum capistratum*)  Black-capped chicadee (*Poecile atricapillus)*  Brown fulvetta (*Alcippe brunneicauda*)  California condor (*Gymnogyps californianus*)  Cape petrel (*Daption capense*)  Carolina chickadee (*Poecile carolinensis*)  Cassin’s auklet (*Ptychoramphus aleuticus*)  Chestnut-winged babbler (*Stachyris erythroptera*)  Collared flycatcher (*Ficedula albicollis*)  Common eider (*Somateria mollissima*)  Cory’s shearwater (*Calonectris borealis*)  Crested auklet (*Aethia cristatella*)  Eurasian woodcock (*Scolopax rusticola*)  European white stork (*Ciconia ciconia*)  Ferruginous babbler (*Trichastoma bicolor*)  Florida scrub jay (*Aphelocoma coerulescens*)  Fluffy-backed tit-babbler (*Macronus ptilosus*)  Giant petrel (*Macronectes* spp.)  Gouldian finch (*Erythrura gouldiae*)  Greater rhea (*Rhea americana*)  Grey partridge (*Perdix perdix*)  Griffon vulture (*Gyps fulvus*)  Hairybacked bulbul (*Tricholestes criniger*)  Hawaii amakihi (*Chlorodrepanis virens*)  Horsfield's babbler (*Malacocincla sepiaria*)  Jackdaw (*Corvus monedula*)  Large-billed crow (*Corvus macrorhynchos*)  Lesser kestrel (*Falco naumanni)*  Little spiderhunter (*Arachnothera longirostra*)  Macaron penguin (*Eudyptes chrysolophus*)  Mallard duck (*Anas platyrhynchos*)  Mourning dove (*Zenaida macroura*)  Northern cardinal (*Cardinalis cardinalis*)  Northern spotted owl (*Strix occidentalis caurina*)  Northern wheatear (*Oenanthe oenanthe*)  Nuttall's white-crowned sparrow (*Zonotrichia leucophrys nuttalli*)  Pine siskin (*Spinus pinus*)  Red kite (*Milvus milvus*)  Red knot (*Calidris canutus*)  Rufous-collared sparrow (*Zonotrichia capensis*)  Rufous-crowned babbler (*Malacopteron magnum*)  Rufous-necked snow finch (*Pyrgilauda ruficollis*)  Short-tailed babbler (*Malacocincla malaccensis*)  Snow goose (*Anser caerulescens*)  Spotless starling (*Sturnus unicolor*)  Superb starling (*Lamprotornis superbus*)  Thrush nightingale (*Luscinia luscinia*)  Wandering albatross (*Diomedea exulans*)  White-ruffed manakin (*Corapipo altera*)  White-rumped snow finch (*Onychostruthus taczanowskii*)  Wood duck (*Aix sponsa*) | 1 |
| Mammals | Cow (*Bos taurus*)  Rat (*Rattus* spp.) | 11 |
|  | Rhesus macaque (*Macaca mulatta*) | 8 |
|  | African striped mice (*Rhabdomys pumilio*)  Pig (*Sus scrofa domesticus*) | 7 |
|  | Western lowland gorilla (*Gorilla gorilla gorilla*) | 6 |
|  | Chimpanzee (*Pan troglodytes*)  Degu (*Octodon degus*)  Sheep (*Ovus* spp.) | 4 |
|  | Baboon (*Papio cynocephalus*)  Cheetah (*Acinonyx jubatus*)  Dog (*Canis familiaris*)  Koala (*Phascolarctos cinereus*)  Mandrill (*Mandrillus sphinx*)  Mice (*Mus* spp.)  Richardson’s ground squirrel (*Spermophilus richardsonii*)  Spotted hyena (*Crocuta crocuta*) | 3 |
|  | Arctic ground squirrel (*Spermophilus parryii*)  Banded mongoose (*Mungos mungo*)  Capuchin monkey (*Sapajus libidinosus*)  Grizzly bear (*Ursus arctos horribilis*)  Horse (*Equus caballus*)  Northern elephant seal (*Mirounga angustirostris*)  Orangutan (*Pongo pygmaeus*)  Red deer (*Cervus elaphus*)  Ring-tailed lemur (*Lemur catta*)  Talas tuco-tuco (*Ctenomys talarum*) | 2 |
|  | African elephant (*Loxodonta africana*)  Agile gracile opossum (*Gracilinanus agilis*)  Alpine chipmunk (*Tamias alpinus*)  Alpine ibex (*Capra ibex ibex*)  American pika (*Ochotona princeps*)  Assanese macaque (*Macaca assamensis*)  Barbary macaque (*Macaca sylvanus*)  Barrow Island euro (*Macropus robustus isabellinus*)  Bison (*Bison bison bison*)  Black bear (*Ursus americanus*)  Black capuchin **(***Sapajus nigritus)*  Black howler monkey (*Alouatta pigra*)  Black tufted-ear marmoset (*Callithrix penicillata*)  Black-footed ferret (*Mustela nigripes*)  Blue monkey (*Cercopithecus mitis*)  Bornean orangutan (*Pongo pygmaeus wurmbii*)  Brush-tailed bettong (*Bettongia penicillata*)  Cabrera vole (*Iberomys cabrerae*)  Cairo spiny mouse (*Acomys cahirinus*)  California mouse (*Peromyscus californicus*)  California sea lion (*Zalophus californianus*)  Collared brown lemur (*Eulemur collaris*)  Common marmoset (*Callithrix jacchus*)  Coquerel's sifaka (*Propithecus coquereli*)  Cotton‐top tamarin (*Saguinus oedipus*)  Crested macaque (*Macaca nigra*)  Daubenton’s bat (*Myotis daubentonii*)  Dingo (*Canis dingo*)  Dromedary camel (*Camelus dromedarius*)  Dugong (*Dugong dugon*)  Edible dormice (*Glis glis*)  Elephant seals (*Mirounga* spp.)  Fishing bat (*Myois vivesi*)  Geladas (*Theropithecus gelada*)  Gemsbok (*Oryx gazelle gazella*)  Giraffe (*Giraffa camelorpadalis giraffa*)  Grey mouse lemur (*Microcebus murinus*)  Guanacos *(Lama guanicoe*)  Guinea pig (*Cavia porcellus*)  Harbor seals (*Phoca vitulina*)  Hawaiian monk seal (*Monachus schauinslandi*)  Highveld mole-rat (*Cryptomys hottentotus pretoriae*)  Iberian lynx (*Lynx pardinus*)  Japanese macaque (*Macaca fuscata*)  Killer whale (*Orcinus orca*)  Leopard (*Panthera pardus*)  Lodgepole chipmunk (*Tamias speciosus*)  Long-Evans hooded rat (*Rattus norvegicus domestica*)  Marmoset (*Callithrix geoffroyi*)  Meerkat (*Suricata suricatta*)  Mongolian gerbil (*Meriones unguiculatus*)  Moose (*Alces alces*)  Mountain gorilla (*Gorilla beringei beringei*)  Mule deer (*Odocoileus hemionus*)  Muskoxen (*Ovibos moschatus*)  New Zealand white rabbit (*Oryctolagus cuniculus*)  North American red squirrel (*Tamiasciurus hudsonicus*)  Olive baboon (*Papio anubis*)  Pig‐tailed Macaque (*Macaca nemestrina*)  Plains zebra (*Equus quagga*)  Przewalski’s horse (*Equus ferus przewalskii*)  Short-tailed fruit bat (Carollia perspicillata)  Siberian hamster (*Phodopus sungorus)*  Snowshoe hare (*Lepus americanus*)  Social tuco-tuco (*Ctenomys sociabilis*)  South American fur seal (*Arctocephalus australis*)  Squirrel monkey (*Saimiri sciureus*)  Steller sea lion (*Eumetopias jubatus*)  Taiwanese pangolin (*Manis pentadactyla pentadactyla*)  Vampire bat (*Desmodus rotundus*)  Vervet monkey (*Chlorocebus aethiops sabaeus)*  Weddell seal (*Leptonychotes weddellii*)  Western grey kangaroo (*Macropus fuliginosus*)  White faced capuchin (*Cebus capucinus)*  White-lipped peccary (*Tayassu pecari*)  White-tailed deer (*Odocoileus virginianus*)  Wildebeest (*Connochaetes taurinus*)  Yellow bellied marmot (*Marmota flaviventris*) | 1 |
